# Phylogenetic and chromosomal analyses of multiple gene families syntenic with vertebrate Hox clusters

**DOI:** 10.1186/1471-2148-8-254

**Published:** 2008-09-19

**Authors:** Görel Sundström, Tomas A Larsson, Dan Larhammar

**Affiliations:** 1 Department of Neuroscience, Uppsala University, Box 593, 75124 Uppsala, Sweden

## Abstract

**Background:**

Ever since the theory about two rounds of genome duplication (2R) in the vertebrate lineage was proposed, the Hox gene clusters have served as the prime example of quadruplicate paralogy in mammalian genomes. In teleost fishes, the observation of additional Hox clusters absent in other vertebrate lineages suggested a third tetraploidization (3R). Because the Hox clusters occupy a quite limited part of each chromosome, and are special in having position-dependent regulation within the multi-gene cluster, studies of syntenic gene families are needed to determine the extent of the duplicated chromosome segments. We have analyzed in detail 14 gene families that are syntenic with the Hox clusters to see if their phylogenies are compatible with the Hox duplications and the 2R/3R scenario. Our starting point was the gene family for the NPY family of peptides located near the Hox clusters in the pufferfish *Takifugu rubripes*, the zebrafish *Danio rerio*, and human.

**Results:**

Seven of the gene families have members on at least three of the human Hox chromosomes and two families are present on all four. Using both neighbor-joining and quartet-puzzling maximum likelihood methods we found that 13 families have a phylogeny that supports duplications coinciding with the Hox cluster duplications. One additional family also has a topology consistent with 2R but due to lack of urochordate or cephalocordate sequences the time window when these duplications could have occurred is wider. All but two gene families also show teleost-specific duplicates.

**Conclusion:**

Based on this analysis we conclude that the Hox cluster duplications involved a large number of adjacent gene families, supporting expansion of these families in the 2R, as well as in the teleost 3R tetraploidization. The gene duplicates presumably provided raw material in early vertebrate evolution for neofunctionalization and subfunctionalization.

## Background

The presence of several paralogous gene regions, forming so-called paralogons [1] with gene family members on two to four chromosomes in mammals, has been taken as evidence for two rounds of whole genome duplication "2R", deduced to have taken place in the ancestor of vertebrates [1-11]. The Hox gene clusters (located on human chromosomes 2, 7, 12 and 17) have been used as the prime example of quadruplicate paralogy in vertebrate genomes, reviewed by Hoegg and Meyer [12], as compared to the single Hox cluster in the cephalochordate lineage [13]. Until recently the number of Hox clusters in cartilaginous fishes has been unclear. The sequencing of the elephant shark genome (*Callorhinchus milii*) established that four Hox-clusters were already present in the ancestor of jawed vertebrates [14]. The Hox clusters are also known to be special in many ways, particularly in the way they are transcriptionally regulated [15]. Tunicate species like *Ciona intestinalis *and *Oikopleura dioika*, which have broken Hox clusters [16-18], are considered to be exceptions from the linear organization of vertebrate and cephalochordate Hox clusters, because the linear cluster of Hox genes was present in the last common ancestor of all bilaterians and possibly before the origin of Cnidarians [15,19].

In ray-finned fishes, an additional tetraploidization (3R) has been inferred based on the occurrence additional Hox clusters not present in other vertebrate lineages [12,20-22]. This has been confirmed using whole genome data from fully sequenced teleosts [23-27]. The study of Hox genes as well as other gene families in several species of actinopterygians has further indicated that the duplication event took place early in the evolution of actinopterygians [22,28]. It has also been proposed that the high number of duplicate genes created in 3R was in part involved in the radiation of teleost fishes [28-30]. The great number of species within the Euteleostei as compared to more basal actinopterygians supports the idea that genome duplication and speciation are linked [24,28] even though there seem to be a large time span between the duplication event and the actual radiation of euteleosts [31,32]. Several teleost lineages, for example Cyprinidae, Salmonidae, and Catostomidae [33-36] are known polyploids of relatively recent origin. The relation between polyploidy and speciation is not yet fully clear but it is worth noting that several species-rich fish clades are polyploids [37]. Both the existence of tetraploids and evidence of independent gene duplications indicate that fish genomes are in some sense more "plastic" than genomes in other vertebrates [29].

After duplication, the genes can undergo nonfunctionalization, subfunctionalization or neofunctionalization. It has been calculated that a duplicated gene in general has an average half-life of approximately 4 million years before it will be silenced [38]. But there are also examples of genes that have recently become pseudogenes even if the duplication took place a long time ago. Some of those examples are the Hoxb7 gene in *Takifugu rubripes *[39] and many of the olfactory genes in hominids [40]. Another example is the neuropeptide Y receptor Y6 that arose very early in vertebrate evolution and later has been mutated to become a pseudogene seemingly independently in many mammalian lineages [41]. The rate of retention of genes after duplication has been investigated in several lineages. The obtained values vary considerably depending on species and time since tetraploidization, for example 8–16% in yeast [42-44], 15–24% for teleosts after 3R [25,45,46], 30% in *Arabidopsis *after its last tetraploidization [47] and about 50% after the salmonid tetraploidization [30]. It also seems like there is (at least in *Arabidopsis*) a connection between "survival" after the first and any subsequent genome duplications. Genes that have been preserved after the first tetraploidization are more likely to be retained after subsequent tetraploidization [48]. However, several factors obscure analyses of old duplication events, such as the frequent loss of genes after duplication, gene conversion and unequal evolutionary rates of paralogs. Because of this the use of the topology of phylogenetic trees as the only criterion for inferring block duplications is not sufficient and the symmetrical (A, B), (C, D) topology expected by two duplication events is rarely seen [9,49]. We believe that a combination of phylogenetic and map-based approaches using several species is much more useful to resolve the evolutionary history of gene families as has been pointed out earlier [50,51]. Although there is evidence for at least one tetraploidization in early vertebrate evolution the concept of two basal vertebrate tetraploidizations has been difficult to establish until recent studies that used both phylogenetic and positional information [11] or only the latter [10] from several sequenced vertebrate genomes. The recent publication of the amphioxus genome confirmed quadruplication of gene regions in gnathostomes [52].

Because each Hox cluster is located in a rather limited part of its chromosome, studies of more gene families are needed to fully understand the evolutionary history of the Hox-bearing chromosomes. In this work we have performed phylogenetic analyses of gene families located near the Hox clusters in several vertebrates in order to see if there are additional gene families with an evolutionary history similar to that of the Hox clusters. Our starting point in this work was the position of the genes coding for neuropeptide Y (NPY) family peptides in the pufferfish *Takifugu rubripes *and the zebrafish *Danio rerio *compared to the positions in the human genome.

## Results

**Figure 1:**
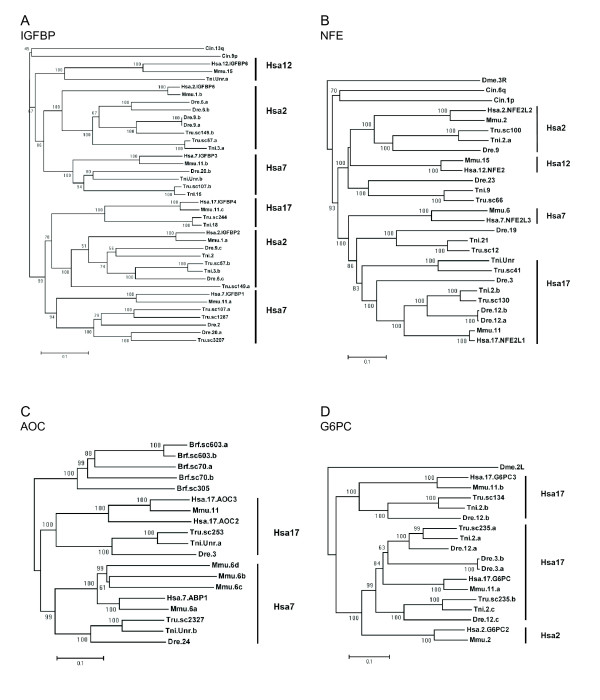
**A-D – Phylogenetic trees for four of the investigated gene families**. Phylogenetic neighbor-joining trees for four of the gene families analyzed in this study (A: IGFBP, B: NFE, C: AOC and D: G6PC). All trees constructed with the NJ method as implemented in Clustal W 1.81 with 1000 bootstrap replicates (values shown for each node). For IGFBP, sequences without the domains PF00219 (Insulin-like growth factor binding protein) and PF00086 (Thyroglobulin type-1 repeat) were removed from the alignment. Note that the tree contains two subfamilies relevant for this paralogon. Hsa is human (Homo sapiens), Mmu: Mouse (Mus musculus), Dre: zebrafish (Danio rerio), Tru: fugu (Takifugu rubripes), Tni: tetraodon (Tetraodon nigroviridis), Cin: (Ciona intestinalis), Brf (Branchiostoma floridae) and Dme: (Drosophila melanogaster). For complete phylogenetic analysis of all 14 families see Additional files 1 and 2.

In total, 14 protein families predicted by the Ensembl database were studied in detail (see Fig. 1A–D and Additional files 1 and 2). Some of these families were further divided into subfamilies based on positions of invertebrate sequences in the initial phylogenetic analysis. Species included in the initial analysis were chosen in order to achieve relative dating of the duplications, thus avoiding analyses of duplication events that occurred before the emergence of chordates (see methods).

Three families in addition to the 14 analyzed families fulfilled the selection criteria (see methods) but had to be excluded for technical reasons, namely because repeated domains made the sequences difficult to align, thereby precluding reliable phylogenetic analyses. These families were ASB (ankyrin-repeat proteins with a SOCS box), HDAC (histone deacetylase) and MGAT (mannoside acetylglucosaminyltransferase).

**Figure 2:**
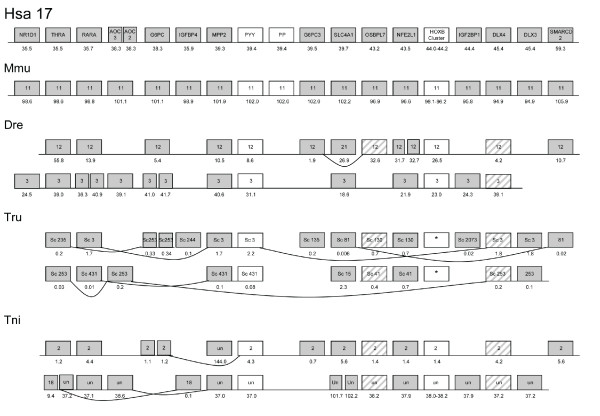
**Conserved synteny between human chromosome 17 and chromosomes of other vertebrate species**. Picture illustrating conservation of synteny between the species included in this analysis. Gene order is depicted based on the positions in the human genome. Genes located on the same chromosomes or scaffolds in other species are connected with lines in the figure. The names of the gene families are given in the boxes representing the human genes and chromosome number or scaffold number is given in the boxes representing the mouse and fish genes. Numbers below boxes indicate chromosomal or scaffold position in megabases. Two narrow boxes next to each other indicate local duplications. White boxes represent gene families not analyzed with phylogenetic methods in this study (NPY and Hox). Striped boxes indicate genes for which phylogeny is inconclusive but where the positional information is in agreement with the proposed paralogon. Boxes denoted "*" for *Takifugu rubripes *Hox clusters indicates that the Hox cluster is scattered on multiple scaffolds, due to incomplete assembly of the genome. Note that two separate parts of *Tetraodon nigroviridis *chromosome 2 displays conserved synteny with human chromosome 2 and 17.

**Figure 3:**
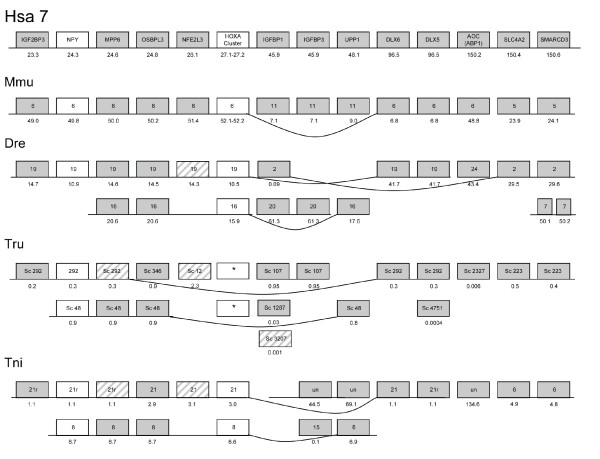
**Conserved synteny between human chromosome 7 and chromosomes of other vertebrate species**. Gene order, gene name, position, local duplications and linkage of genes are indicated in the same way as in Fig. 2.

**Figure 4:**
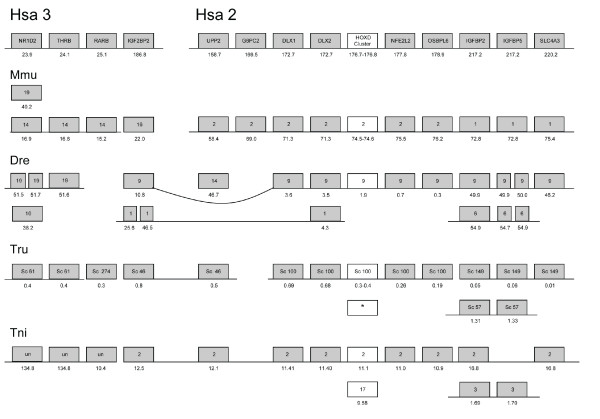
**Conserved synteny between human chromosomes 2/3 and chromosomes of other vertebrate species**. Gene order, gene name, position, local duplications and linkage of genes are indicated in the same way as in Fig. 2.

**Figure 5:**
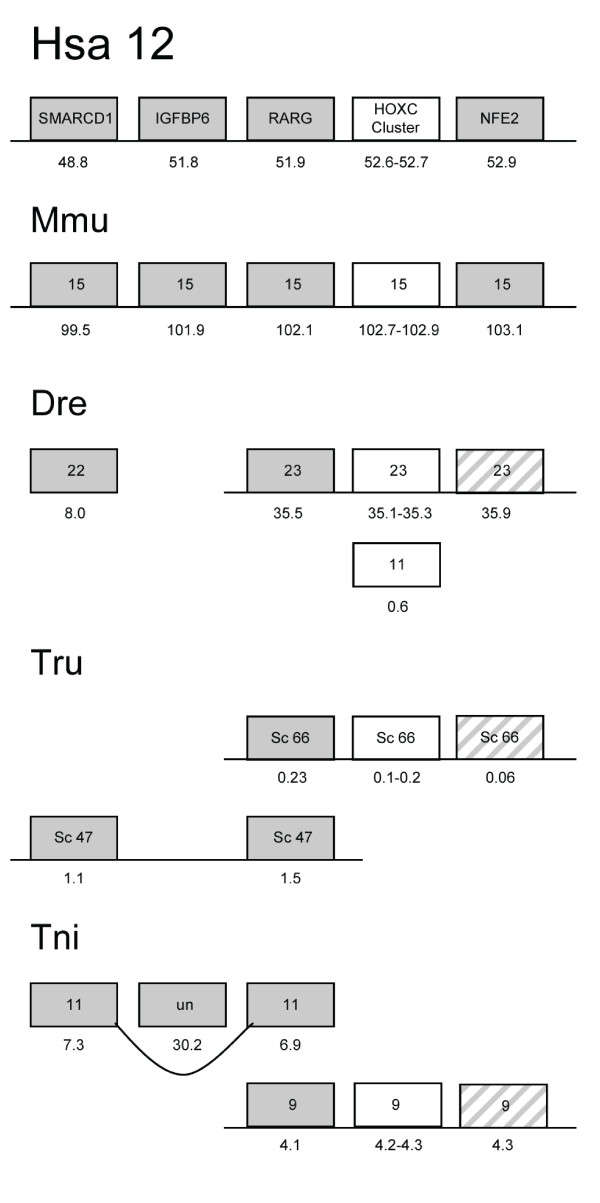
**Conserved synteny between human chromosome 12 and chromosomes of other vertebrate species**. Gene order, gene name, position, local duplications and linkage of genes are indicated in the same way as in Fig. 2.

For some of the fish sequences no clear human orthologs are found in the phylogenetic trees (one such example is the NR1D tree where the fish genes are clustered together without any tetrapod sequence). However, when chromosome position information is taken into account it becomes apparent that there is conserved synteny between fish and human chromosomes indicated by striped boxes in Figs. 2, 3, 4, 5. For a majority of the phylogenetic trees the topologies in the NJ and ML analysis are in agreement (see Additional files 1 and 2).

### Gene families represented on four human Hox chromosomes

*IGFBP*, insulin like growth factor binding proteins, are part of a loosely defined superfamily [53]. The IGFBP family studied here is comprised by the six members with high-affinity binding to insulin and is represented on all four of the human HOX-bearing chromosomes. On both Hsa 2 and 7 they appear as gene pairs resulting from a duplication before the separation of the branch leading to the mammals and the branch leading to the teleosts (see discussion). Both of these pairs display retained 3R copies in at least one fish species for each gene (Fig. 1A). One *Takifugu rubripes *gene, called Tru.sc149.a in Fig. 1A, appears in an unexpected position in the tree. However, this sequence has a deviating segment compared to all other IGFBP sequences, probably due to difficulties with exon-intron prediction in the database. In our quartet puzzling maximum likelihood analysis (Additional file 2), this gene product clusters with the *Tetraodon nigroviridis *sequence Tni.2, favoring orthology in agreement with the chromosomal position (Fig. 2). This family has recently been studied by others [54] giving a similar topology but with a different interpretation of the evolutionary history of this gene family (see discussion).

*NFE2 *(Nuclear factor erythroid 2) family members are involved in gene transcription and due to the presence of four genes located in the vicinity of all four Hox clusters, this family has previously been suggested to have had a similar evolutionary history as the Hox clusters [55,56]. Our results are compatible with such a scenario but the topology in our tree is somewhat difficult to interpret (Fig. 1B), especially regarding identification of fish orthologs. This is probably due to problems with obtaining a reliable alignment for this family because the paralogs only have a short conserved domain in otherwise divergent sequences. Nevertheless, all genes display conserved synteny and have evolved in a time frame consistent with 2R.

### Gene families represented on three human Hox chromosomes

*DLX *genes form gene pairs on three of the chromosomes (2, 7 and 17) in the Hox paralogon. Three of these human genes (one on each chromosome) cluster in the phylogenetic trees with genes that were duplicated in fishes (Additional files, figures 1B and 2B). The Dlx family is a widely studied family with important functions in vertebrate development [57]. Our results confirm an early local duplication followed by chromosome duplication for this family as previously suggested [58-61].

*IGF2BP*, IGF-2-mRNA binding proteins, also called IMPs, are part of an mRNA localization and transport system. The family has members on three of the human chromosomes analyzed in the study. It has previously been suggested that the family originated by duplications before the vertebrate radiation [62,63]. Our phylogenetic analyses support an origin in 2R and also an expansion in the zebrafish in 3R (Additional files, figures 1E and 2E).

*SLC4A *is a family that is part of the solute carrier superfamily and catalyzes transmembrane bicarbonate transport in the anion exchanger (AE) subfamily [64]. Others have recently analyzed a larger part of this superfamily resulting in a similar tree topology [54]. The family has members on Hsa 2, 7 and 17 and as in many of the other gene families, only one case where both copies resulting from 3R have been retained (Additional files, figures 1K and 2K).

*SMARCD*, SWI/SNF-related, matrix-associated, actin-dependent regulators of chromatin (SMARC) is a family involved in chromatin remodeling complexes [65]. We have studied one subfamily with three human members (located on chromosomes 7, 12 and 17). It seems like a lineage-specific duplication has occurred in *Ciona*, but the rest of the family has expanded in a time window in agreement with 2R (Additional files, figures 1L and 2L).

**Figure 6:**
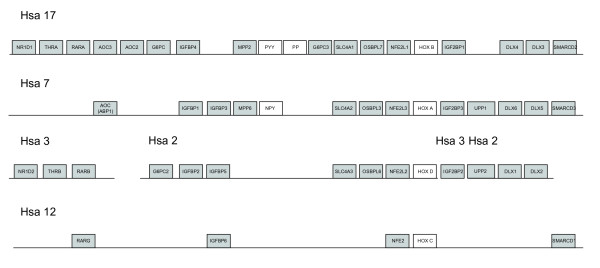
**Schematic picture of human paralogs**. Schematic picture illustrating the human paralogon analyzed in this study. Gene order as on chromosome 17. Observe that figure is not drawn to scale.

*OSBPL*, Oxysterol-binding proteins are a large family involved in diverse cellular processes with an oxysterol binding domain as common feature [66,67] and we have studied one subfamily with the human members located on chromosomes 2, 7 and 17 (Fig. 6). In one case 3R is clearly visible in the tree (Additional files, figures 1I and 2I).

*RAR*, Retinoic acid receptors, a gene family belonging to the nuclear receptor superfamily (see below).

### Gene families represented on two human hox chromosomes

#### Nuclear receptors

The gene families belonging to the nuclear receptor superfamily, RAR, THR and NR1D, have been studied previously [68,69] with similar results as obtained in this study. The NR1D and THR families have members on Hsa 3 and 17, and the RAR family also includes a member on Hsa 12. Some duplicates from 3R are also retained (Additional files, figures 1H, J and 1M and 2H, J and 2M) and these display conserved synteny with their mammalian orthologs (Fig. 2, 4 and 5). It should be noted that the timing for the duplications is uncertain in the NR1D family with one *Branchiostoma floridae *sequence clustering together with orthologs of the vertebrate NR1D1 sequences, however with low bootstrap support (see Additional files, figures. 1H and 2H).

*AOC*, (amine oxidase) gene family (Fig. 1C) belongs to the copper-binding amino oxidase superfamily. The human members in this study include one on chromosome 7 and two on chromosome 17. The chromosomal locations are as expected by block duplication in early vertebrate evolution (Fig. 1C and Additional files, figures 1A and 2A).

*G6PC *(glucose-6-phospatase beta) (Fig. 1D) is an enzyme involved in both gluconeogenesis and glycogenolysis and has two members on Hsa 17 and one on Hsa 2 [70]. Due to lack of sequences from Ciona and *Branchiostoma floridae*, the time window when the expansion of this family took place is wide. The duplication on Hsa 17 is present also in the fishes, indicating origin before the divergence of sarcopterygians and actinopterygians. In addition, the teleost fishes have a local duplication of one of these orthologs resulting in three members of this family located on the same chromosome (Fig. 2). For one of these genes it seems like the zebrafish has retained both of the copies formed in 3R (Fig. 1D and Additional files, figures 1C and 2C). Furthermore, the Dre3 gene has undergone a recent duplication in the zebrafish (Fig. 1D), although this could also be a result of an assembly error in the database.

*MPP*, is a subfamily of the membrane-associated guanylase kinase homologs superfamily, an old family involved in cell signaling [71]. Our subfamily has members on Hsa 7 and 17, the tree displays a topology consistent with 3R (Additional files, figures 1F and 2F).

*UPP*, uridine phosphorylate is a small family that catalyzes the reversible phosphorylitic cleavage of uridine, deoxyuridine and thymidine [72]. It consists of only two human members that seem to have evolved in the same time frame as the proposed 2R events (Additional files, figures 1N and 2N).

## Discussion

Extending our previous observation that the two NPY-family genes NPY and PYY are located near the Hox clusters [73], we recently reported that these syntenies exist also in the pufferfishes *Takifugu rubripes *and *Tetraodon nigroviridis*, the medaka *Oryzias latipes*, the stickleback *Gasterosteus aculeatus *and the zebrafish *Danio rerio *[74]. Thus, these syntenies are likely to have existed in the common ancestor of tetrapods (and other sarcopterygians) and actinopterygians. These chromosome regions have undergone further duplications in the teleost fish tetraploidization, 3R, resulting in duplicates of both the Hox clusters and the NPY and PYY genes. Several other gene families have been suggested to have been duplicated together with the Hox clusters [7-9,54]. To our knowledge the evolutionary histories of only a few families have been investigated in the Hox paralogon using both sequence phylogenies and synteny comparisons, for example the DLX family [58,59], the nuclear receptor family [68] and the voltage-gated sodium channels [75,76]. In the present study, we have investigated in detail several additional gene families that are represented on multiple chromosomes in the Hox paralogon. With the NPY-family genes as starting points in *Takifugu rubripes*, *Danio rerio *and human, we identified 14 gene families that are present on two, three, or all four of the human Hox chromosomes. We have studied the phylogenies of these gene families initially with the neighbor-joining method and subsequently with quartet puzzling maximum likelihood, and report here that 13 of the 14 families have members that seem to have been duplicated in the early stages of vertebrate evolution (see table 1). The remaining family, G6PC, also has a topology consistent with 2R but due to lack of urochordate or cephalochordate sequences the time window is wider when these expansions could have occurred. The inclusion of human chromosome 3 as part of this paralogon has been suggested earlier [7,75]. However, it is not clear if paralogs on Hsa3 were ancestrally linked to genes on Hsa2 (see figure 4 and [7]) or Hsa 7 [76] before the duplication events. The current analysis is not able to resolve this ambiguity.

**Table 1:** Summary of the combined interpretation of phylogenetic and positional information

	2R		3R		
Gene family	Phylogenetic support	Comments	Phylogenetic support*	Postional support	Comments
AOC	Yes		No	No	
DLX	Yes		Yes	Yes	Uncertain topology regarding some duplications
G6PC	Yes/No	Uncertain timing, only Drosophila as outgroup	Yes	Yes	Can't exclude lineage specific duplication
IGF2BP	Yes		Yes	Yes	Can't exclude lineage specific duplication
IGFBP	Yes		Yes	Yes	
MPP	Yes		Yes	Yes	Uncertain topology regarding some duplications
NFE	Yes		Yes/No	Yes	Uncertain topology
NR1D	Yes/No	Uncertain topology	No	Yes	Can't exclude lineage specific duplication
OSBPL	Yes		Yes	Yes	Uncertain topology regarding some duplications
RAR	Yes		Yes	Yes	
SLC4A	Yes		Yes	Yes	
SMARCD	Yes		Yes	Yes	Can't exclude lineage specific duplication
THR	Yes		Yes	Yes	
PP	Yes	U	Yes	Yes	Can't exclude lineage specific duplication

* Duplicates in one fish species for one of the mammalian sequences have been considered to be support for 3R.

Two gene families that have members on all four of the human Hox chromosomes were identified, namely IGFBP and NFE2. The NFE2 family sequences are difficult to align as explained in results and thus the phylogenetic analysis must be interpreted with caution, suffice to conclude that the tree topology (Fig. 1B) is consistent with a quadruplication in early vertebrate evolution. From our analysis it is not possible to deduce if the IGFBP family had one or two members at the time of divergence of the common ancestor of actinopterygians and sarcopterygians from the tunicate lineage (Fig. 1A). The local duplication event could have occurred in early vertebrate evolution, whereupon the gene pair was duplicated as a unit, most likely concomitantly with Hox (i.e. in 2R). Two of the IGFBP duplicates seem to have been lost before the divergence of actinopterygians and sarcopterygians. This scenario is one plausible explanation of the present situation in the human genome with IGFBP gene pairs on Hsa 2 and 7 and single genes on Hsa 12 and 17 (see Fig. 6). The most parsimonious explanation for the observed topology is that loss of genes took place between 1R and 2R. Abbasi et al. [54] interpreted this tree differently, stating that the most parsimonious explanation is two genome duplications followed by two local duplications on chromosome 2 and 7 respectively. In our opinion it is not possible to interpret the tree topology this way. If the local duplications occurred after the whole genome duplications one would expect a different topology, with the locally duplicated genes on Hsa7 clustering together and the ones on Hsa2 clustering together. This is not the case (see Fig. 1A). Their alternative hypothesis includes three whole genome duplications and two losses. In order for this scenario to be compatible with the topology and localization two translocations must have occurred. This involves two additional steps and it also suggests that three whole genome duplications have occurred, but this is not observed for any of the other families in their study.

Six gene families were found to be represented on three of the human Hox chromosomes, i.e., DLX, IGF2BP, SLC4A, SMARCD, OSBPL and RAR. Note that the DLX family underwent a local duplication before the chromosome duplications, in analogy with the IGFBP family described above, as shown by the phylogenetic tree in Fig. 1C and the chromosome maps in Figs. 2, 3, 4, 5, 6. This family has previously been difficult to resolve using sequence analyses in mammals, but thanks to the many fish sequences it can be established that DLX1, 4 and 6 form one series of paralogs and that DLX 2, 3 and 5 form another [58].

Representation on two of the mammalian Hox chromosomes was found for six gene families, namely two of the three nuclear receptor families THR and NR1D, as well as AOC, G6PC, MPP, and UPP. The AOC family (Fig. 1C), for instance, is represented on Hsa7 and Hsa17 with orthologs located in the corresponding regions in the mouse genome. A local duplication has occurred in mammals, resulting in AOC2 and AOC3 on Hsa17, with loss of AOC2 in mouse. There is also a third member located on chromosome 17 – a pseudogene that was not present in version 43 of the Ensembl database [77]. A series of duplications has occurred in the mouse genome resulting in four copies on Mmu6 [78]. In contrast, no additional duplications have occurred among the fish species, not even in 3R.

The G6PC family (Fig. 1D) is also represented on two chromosomes in human (Figs. 2 and 4). The local duplication observed on Hsa17 is present also in the three teleosts studied, showing that it happened before the sarcopterygian-actinopterygian split (Fig. 2). Interestingly, the three teleosts have an additional local duplication of one of these genes, not present in the mammals. In this case, zebrafish seems to have retained a 3R duplicate of one of these genes (Dre12a) which has a copy on Dre3.

These gene families show that local duplications occur frequently and thereby complicate delineation of gene family histories that involve both local and chromosome duplications. To distinguish between locally duplicated genes and gene duplicates resulting from large-scale events such as 2R, the use of several species is crucial. It has recently been suggested that gene families located on the Hox-bearing chromosomes were not duplicated together with Hox based on the observed differences in the topologies [54]. Instead the gene families were proposed to have duplicated in several independent small-scale events and later translocated to the same chromosomes [54]. This suggests independent translocation events before the divergence of actinopterygians and sarcopterygians. In the light of the results from large-scale studies describing duplicated regions in the genomes of extant vertebrates and the reconstruction of ancestral chromosomes [10,11] it seems unlikely that the observed pattern arose by many independent duplications followed by translocations to the same chromosomes. Furthermore, the strict demand for (A, B)(C, D)-topologies as the only criteria to support 2R is to restrictive due to complications such as unequal evolutionary rates, gene conversion and loss of genes after duplication. Thus, trees not showing the perfect (A, B)(C, D)-topology are still compatible with large-scale duplication events if the relative dating of these events agrees with positional information from several species.

It was expected that several of the gene families would display fewer than four members in mammals, and likewise fewer than eight members in the teleosts (Figs. 2, 3, 4, 5). As has been described on numerous previous occasions, gene losses are common after duplications why at least one member is often missing. Striking examples of gene loss are seen in the Hox clusters, where the ancestral set of 14 genes should have resulted in 56 genes after the block quadruplication. However, one of the ancestral Hox genes (Hox14) has lost all four paralogs in mammals after the duplications [79-81], and several other Hox genes have lost 1–3 of the duplicates, resulting in the present number of 39 Hox genes in mammalian genomes. Furthermore, both the pufferfishes and zebrafish seem to have lost a whole Hox cluster, albeit different ones in these two lineages [12]. As shown in Fig. 6, information from families with only two members still gives important information when they are interpreted together to define regions that have been duplicated. If loss of genes from duplicated chromosomal segments occurs independently, positional information from families with two members taken together still gives valuable information.

In some of the gene families the teleost-specific duplicates are only represented in one species (Figs. 2, 3, 4, 5). This made it difficult to distinguish lineage-specific duplications from 3R duplications followed by loss of the duplicate in some lineages. We believe the poor representation of 3R duplicates in our trees is partly a reflection of the strict criteria we used when editing the alignments of sequences from incomplete or poorly annotated genome databases. Because sequences lacking the analyzed domains were excluded, our dataset probably underestimates the real number of family members in the included fish species. Some of the genes lacking domains could be on their way to become pseudogenes, but there could also be problems in the prediction of coding regions in the fish genomes, for example due to short introns in *Tetraodon nigroviridis *[25]. Even though we started our analysis in the fish genomes and therefore should not be biased towards any of the human chromosomes, one of them stands out as more poorly represented than the other three with regard to the number of gene families, namely human chromosome 12. Uneven retention of gene duplicates on different chromosomes resulting from a quadruplication has been observed previously for the paralogon comprised by the human chromosomes 4, 5, 8 and 10 [82] as well as for chromosomes 1, 6, 9 and 19 [83]. This could be the result of more extensive rearrangements on some chromosomes, causing loss of genes for instance by affecting gene regulatory elements.

In studies of gene duplications the dating of the duplication events is of course an important factor. This can be done using molecular clocks [84], ideally calibrated against known speciation events. Dating of duplication events can also be obtained by using information from several species, thereby making it possible to relatively date the origin of genes in relation to speciation events. The 2R tetraploidizations have been proposed to have occurred after the split of urochordates and vertebrates but before the origin of gnathostomes [9]. We used sequences from the tunicates *Ciona intestinalis *or *Ciona savignyi *or the cephalochordate *Branchiostoma floridae *as outgroup to see if the duplications we investigated took place in the vertebrate lineage, as the question we wanted to address was whether the syntenic gene families expanded in the same time window. So far it has not been possible to determine unambiguously whether both of the 2R tetraploidizations took place before the origin of cyclostomes, or if the second tetraploidization occurred on the gnathostome branch after it had separated from the cyclostomes mainly due to lack of sequence information.

Many types of genetic events complicate efforts to reconstruct the evolutionary history of individual gene families, not only frequent duplications and losses, but also differences in the rate of change between members in a gene family or in the same gene over time. Another source of difficulties that we have experienced, as mentioned above, is uncertainties in protein alignments due to duplication of domains in some family members. Because sequence-based phylogenetic analyses and chromosome positions (syntenies) constitute two quite different types of information, they can be combined to deduce evolutionary schemes in a more reliable way. The combined use of synteny and phylogenetic information makes it possible to delineate old and new duplications, identify translocations of genes and also allows investigation of gene families with only two members. Mechanisms like crossing-over and gene conversion may still obscure relationships, but at least the latter is expected to decrease with time as the family members accumulate differences that reduce the likelihood of such events. Particularly gene families with very short sequences, and/or highly conserved or rapidly diverging sequences, can be resolved by considering chromosomal position, as has been shown for the insulin/relaxin family of peptides [85], the NPY family of peptides as mentioned above [74], and some of the NPY-family receptors [86] as well as for example the NFE2 family in this study. All of these families were found to have expanded in the early vertebrate tetraploidizations.

## Conclusion

In conclusion, our study shows that the previously well-studied Hox cluster was certainly not the only gene family in this chromosomal segment that was quadrupled in early vertebrate evolution: the duplicated regions extend quite far in both directions from the Hox clusters and involve a larger number of gene families that are totally unrelated to the Hox-gene sequences. Many genes have been lost in these families after the quadruplication, like in the Hox clusters, thereby obscuring the duplication events. Nevertheless, the overall pattern of genes in the regions flanking the four Hox clusters clearly shows that the quadruplication encompasses a major chromosomal segment, probably a complete ancestral chromosome.

## Methods

### Identification of chromosomal regions

The selection of the 14 gene families used in this study was based on the location of family members close to the genes coding for NPY family peptides in fugu, *Takifugu rubripes *and zebrafish, *Danio rerio*. The gene families included have genes located on at least two of the human chromosomes 2, 3, 7, 12 and 17. Chromosome 3 is a non-Hox-bearing chromosome but earlier studies have suggested that it is a part of the Hox-paralogon [7,8,75]. The two gene families IGFBP and SLC4A were included because they were already known to have copies on several of the chromosomes in the paralogon and were located near the human NPY-family genes.

### Database searches

The protein family predictions in Ensembl database version 43 http://www.ensembl.org/ where used together with relevant literature and BLAST [87] to identify additional sequences not included in the Ensembl protein families. All amino acid sequences representing the longest transcripts of every member of the 14 gene families in *Tetraodon nigroviridis*, *Takifugu rubripes*, *Danio rerio*, *Mus musculus *and *Homo sapiens *were obtained in order to obtain information of the repertoires in the common ancestor of actinopterygians and sarcopterygians using the Ensembl database version 43. This allows for the identification of shared and lineage specific in teleosts and tetrapods, respectively. For a full set of Ensembl IDs see Additional file 3.

### Alignments and phylogenetic analyses

Protein domains in each sequence were identified by searches against the Pfam database http://www.sanger.ac.uk/Software/Pfam/ and aligned using the Windows version of Clustal × 1.81 [88,89]. Sequences lacking described domains, or incomplete domains, were removed from the alignment (for description of which criteria that were used for each family see figure legends in supplementary file 1 and 2). Alignments were thereafter manually inspected and edited to remove poorly aligned sequences. Phylogenetic trees were constructed using the neighbor-joining (NJ) method with standard settings (Gonnet weight matrix, gap opening penalty 10.0 and gap extension penalty 0.20) as implemented in the windows version of Clustal W 1.81 [88] with 1000 bootstrap replicates. Bootstrap values below 50% were considered non-supportive. Sequences from *Drosophila melanogaster *and *Ciona intestinalis *(or *Ciona savignyi *or *Branchiostoma floridae*) were used as outgroups in the phylogenetic analyses in order to relatively date duplications. Quartet puzzling maximum-likelihood trees were constructed using Tree-Puzzle 5.2 [90] (for Tree-puzzle settings in each analysis, see supplementary file 2).

### Chromosomal maps

Based on the phylogenetic analyses, positional information from gene family members duplicated after the split of urochordates and the rest of the chordates and before the split of sarcopterygians and actinopterygians was used to draw chromosomal maps. This allowed for identification gene families that most likely were syntenic in the ancestor of all vertebrates.

## Authors' contributions

GS performed the database searches, phylogenetic and chromosome analyses and wrote part of the manuscript. TAL participated in phylogenetic and chromosome analyses and writing of the manuscript. DL initiated the study and participated in its design and coordination and helped to draft the manuscript. All authors read and approved the final manuscript.

## Supplementary Material

Additional file 1 Neighbor-joining trees for the 14 gene families analyzed. Click here for file

Additional file 2 Quartet puzzling trees for the 14 gene families analyzed. Click here for file

Additional file 3 Accession numbers, positional information and description of sequences used in the phylogenetic analyses. Click here for file
