# Supplementary material for: Phylogenetic and chromosomal analyses of multiple gene families syntenic with vertebrate Hox clusters
Source: BMC Evol Biol. 2008 Sep 19;8:254. doi: 10.1186/1471-2148-8-254 (PMC2566581; doi:10.1186/1471-2148-8-254)
Supplement: Additional file 1 — Neighbor-joining trees for the 14 gene families analyzed. [file 1471-2148-8-254-S1.pdf]

Supplementary figure 1A (AOC)

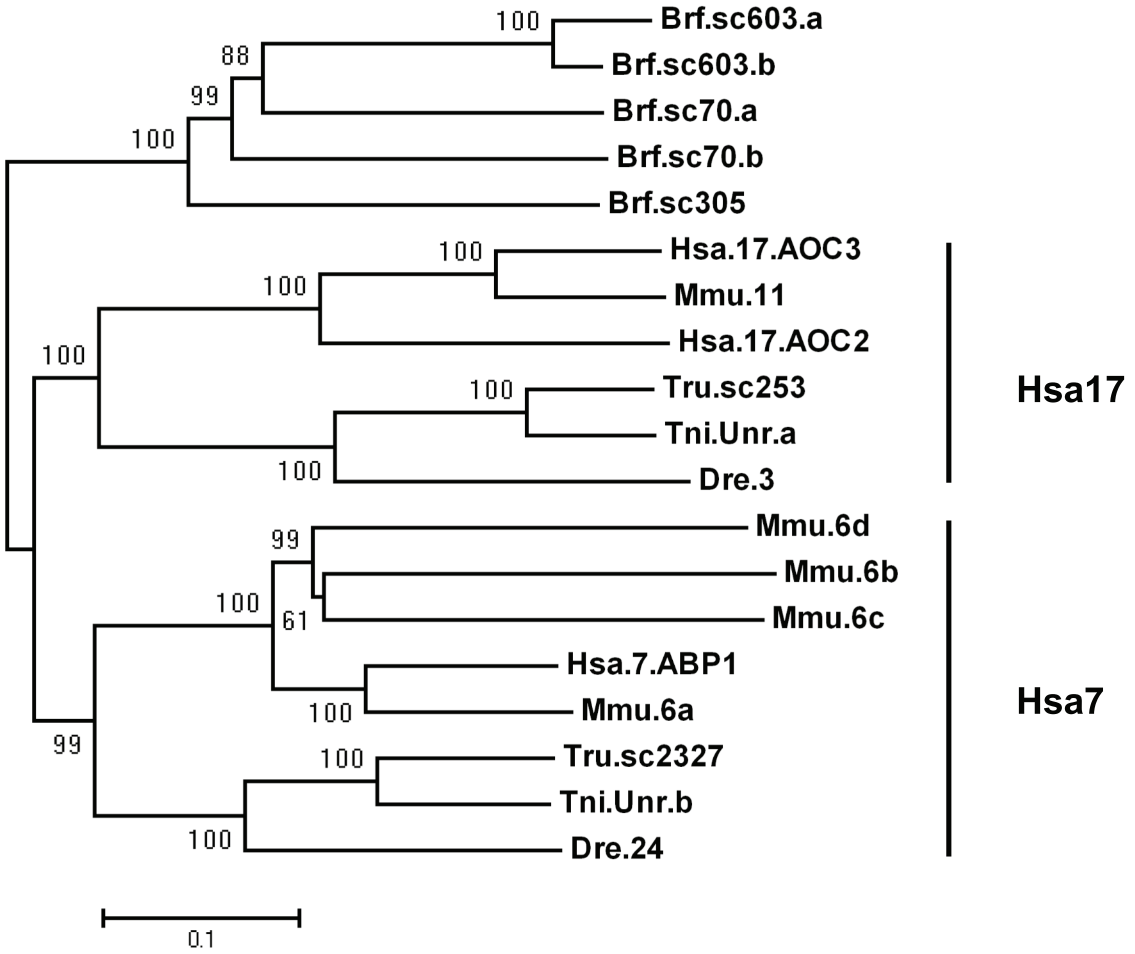

Phylogenetic tree showing the AOC family. Neighbor-joining tree as implemented in ClustalW 1.81 with 1000 bootstrap replicates. Bootstrap percentages shown for each node. Hsa is human (*Homo sapiens*), Mmu: Mouse (*Mus musculus*), Dre: zebrafish (*Danio rerio*), Tru: fugu (*Takifugu rubripes*), Tni: tetraodon (*Tetraodon nigroviridis)* and Brf: amphioxus (*Branchiostoma floridae*).

Supplementary figure 1B (DLX)

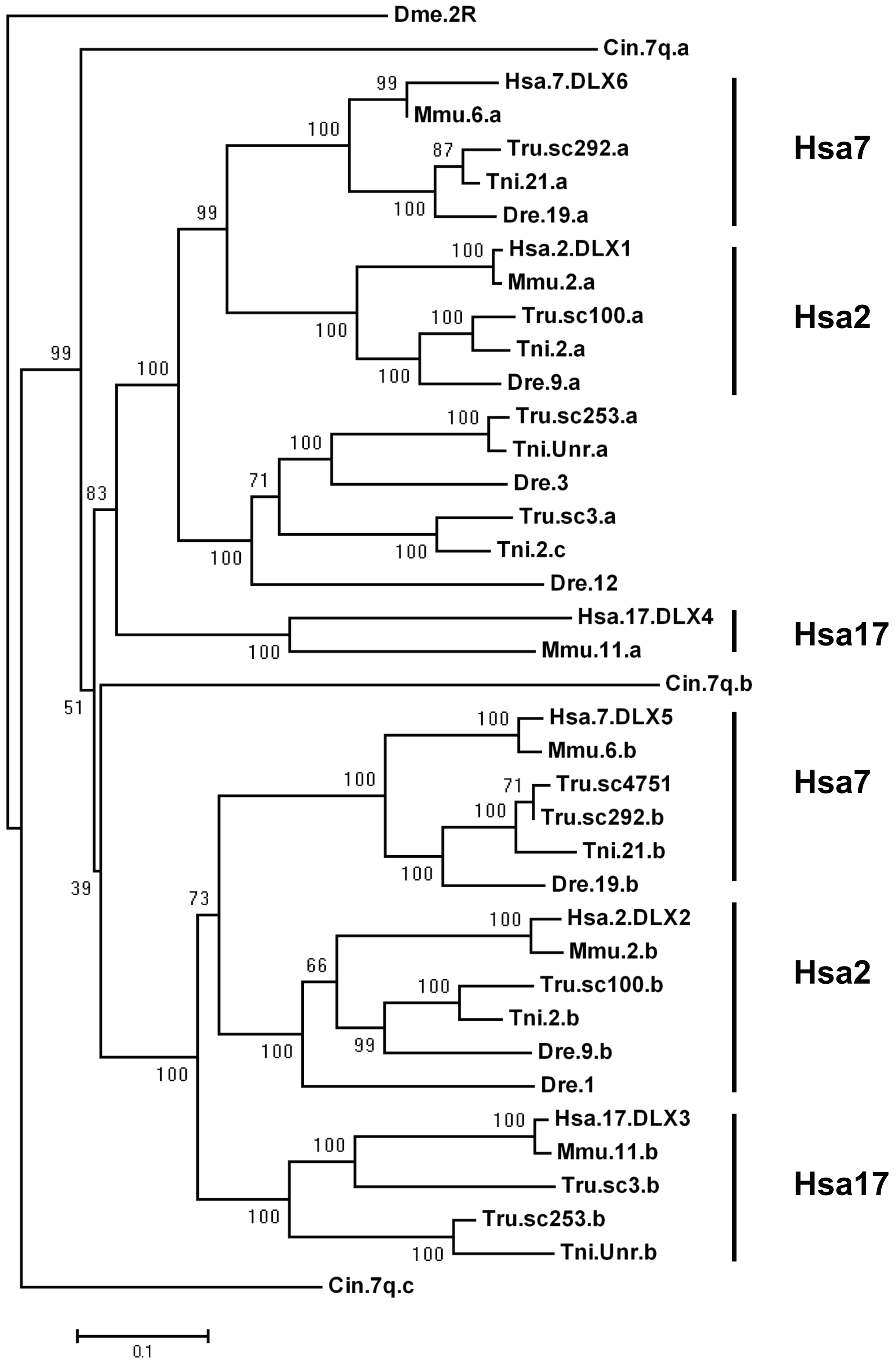

Phylogenetic tree showing the DLX family. Neighbor-joining tree as implemented in ClustalW 1.81 with 1000 bootstrap replicates. Bootstrap percentages shown for each node. Sequences without the homeodomain (pfam ID: PF00046) were removed from the alignment. Abbreviations as in figure 1 A and Cin is *Ciona intestinalis* and Dme: *Drosophila melanogaster*.

Supplementary figure 1C (G6PC)

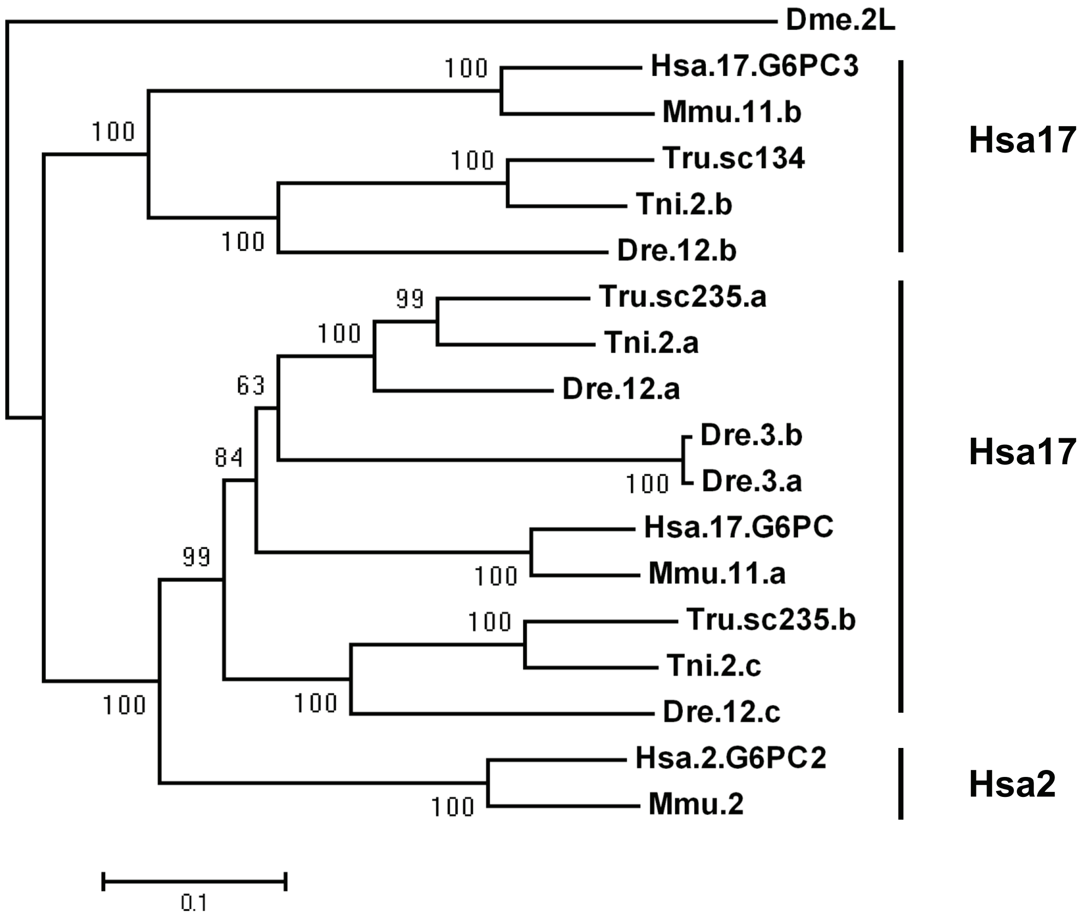

Phylogenetic tree showing the G6PC family. Neighbor-joining tree as implemented in ClustalW 1.81 with 1000 bootstrap replicates. Bootstrap percentages shown for each node. Abbreviations as in previous figures.

Supplementary figure 1D (IGFBP)

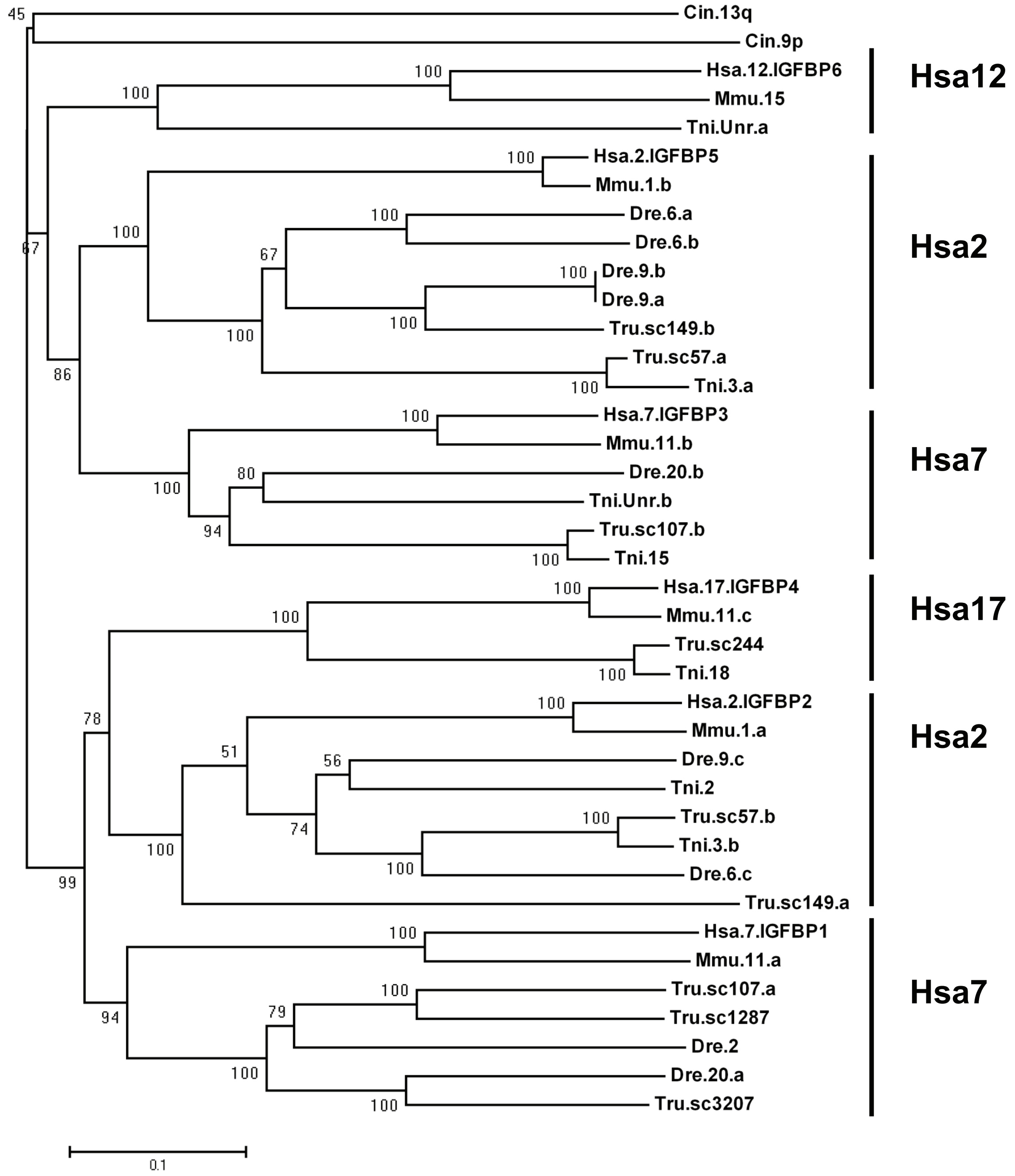

Phylogenetic tree showing the IGFBP family. Neighbor-joining tree as implemented in ClustalW 1.81 with 1000 bootstrap replicates. Bootstrap percentages shown for each node. Sequences without the domains PF00219 (Insulin-like growth factor binding protein) and PF00086 (Thyroglobulin type-1 repeat) were removed from the alignment. Abbreviations as in previous figures. Note that the tree contains two subfamilies relevant for this paralogon.

Supplementary figure 1E (IGF2BP)

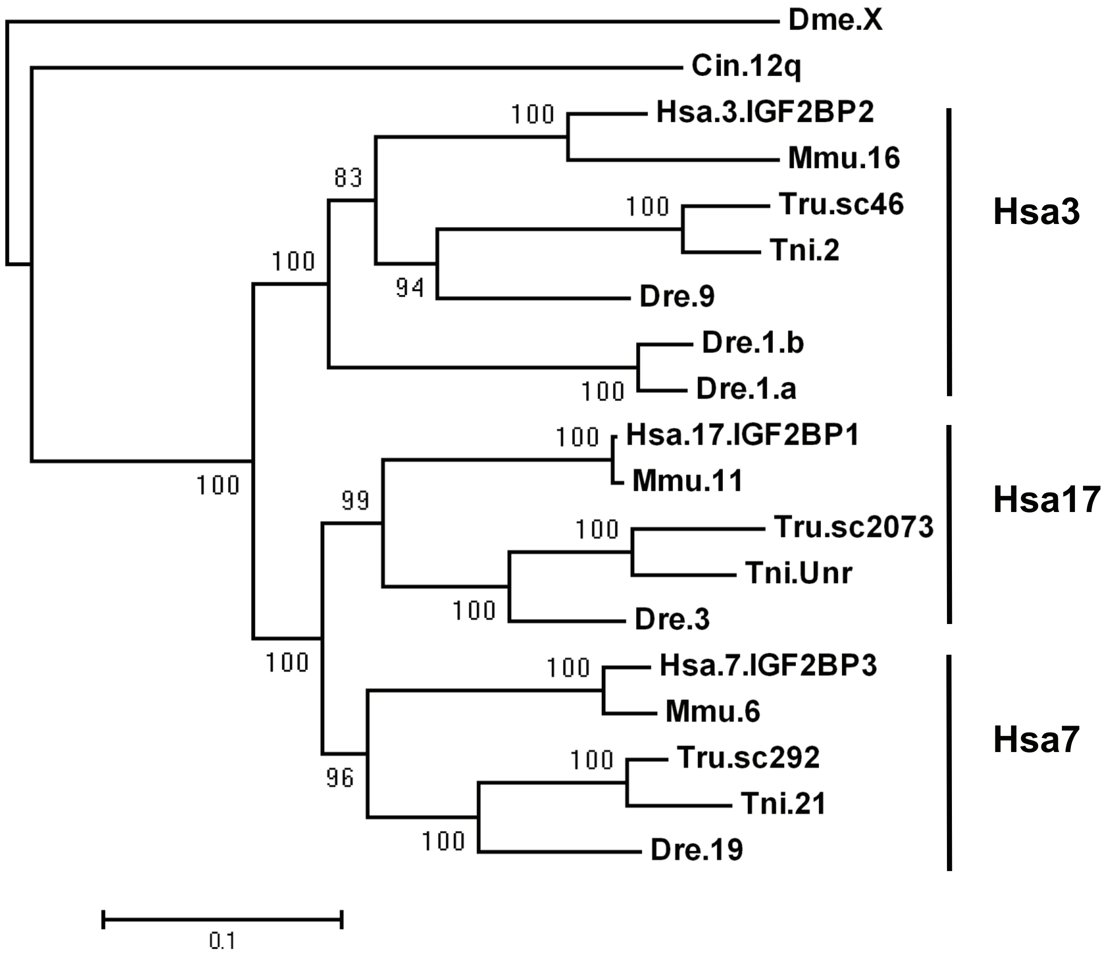

Phylogenetic tree showing the IGF2BP family. Neighbor-joining tree as implemented in ClustalW 1.81 with 1000 bootstrap replicates. Bootstrap percentages shown for each node. Abbreviations as in previous figures.

Supplementary figure 1F (MPP)

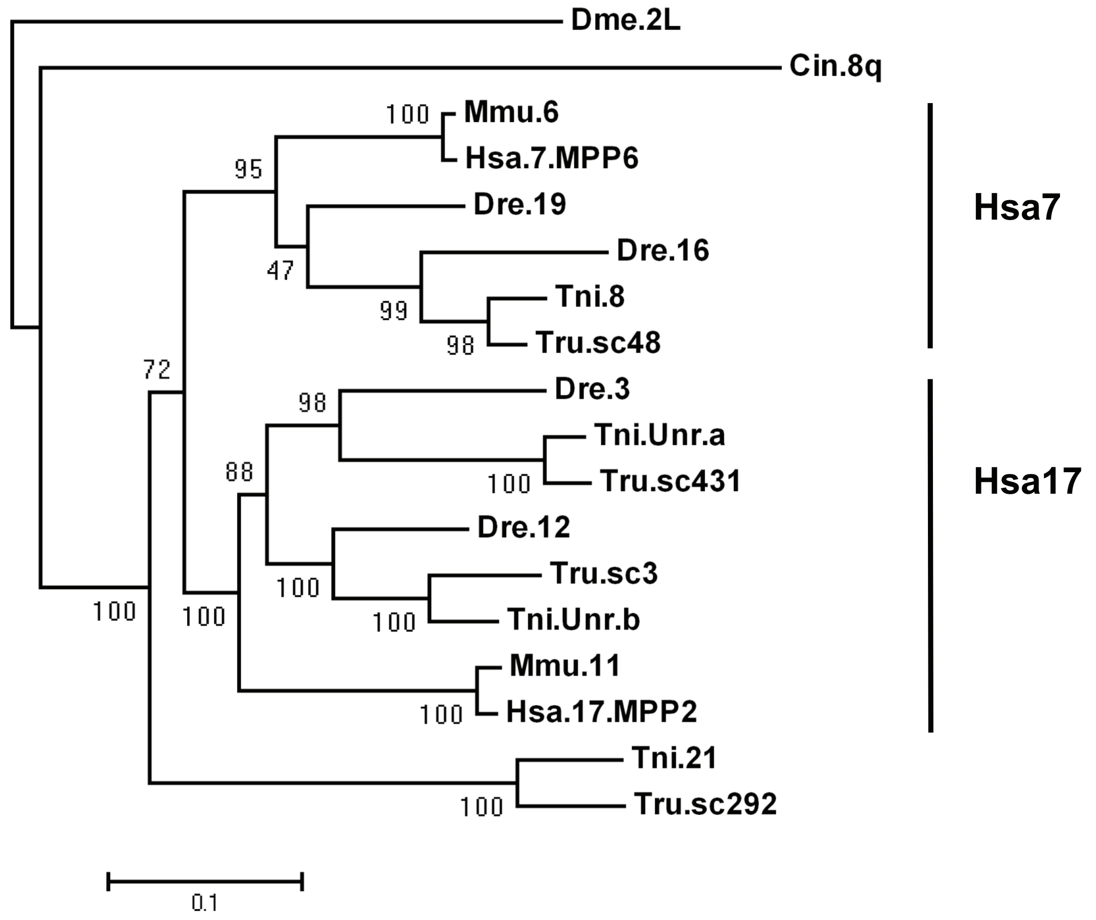

Phylogenetic tree showing the MPP family. Neighbor-joining tree as implemented in ClustalW 1.81 with 1000 bootstrap replicates. Bootstrap percentages shown for each node. Alignment based on the domains PF00595 (PDZ domain), PF00018 (SH3 domain) and PF00625 (Guanylate kinase). Sequences without the domain PF00625 were removed from the alignment. Abbreviations as in previous figures.

Supplementary figure 1G (NFE)

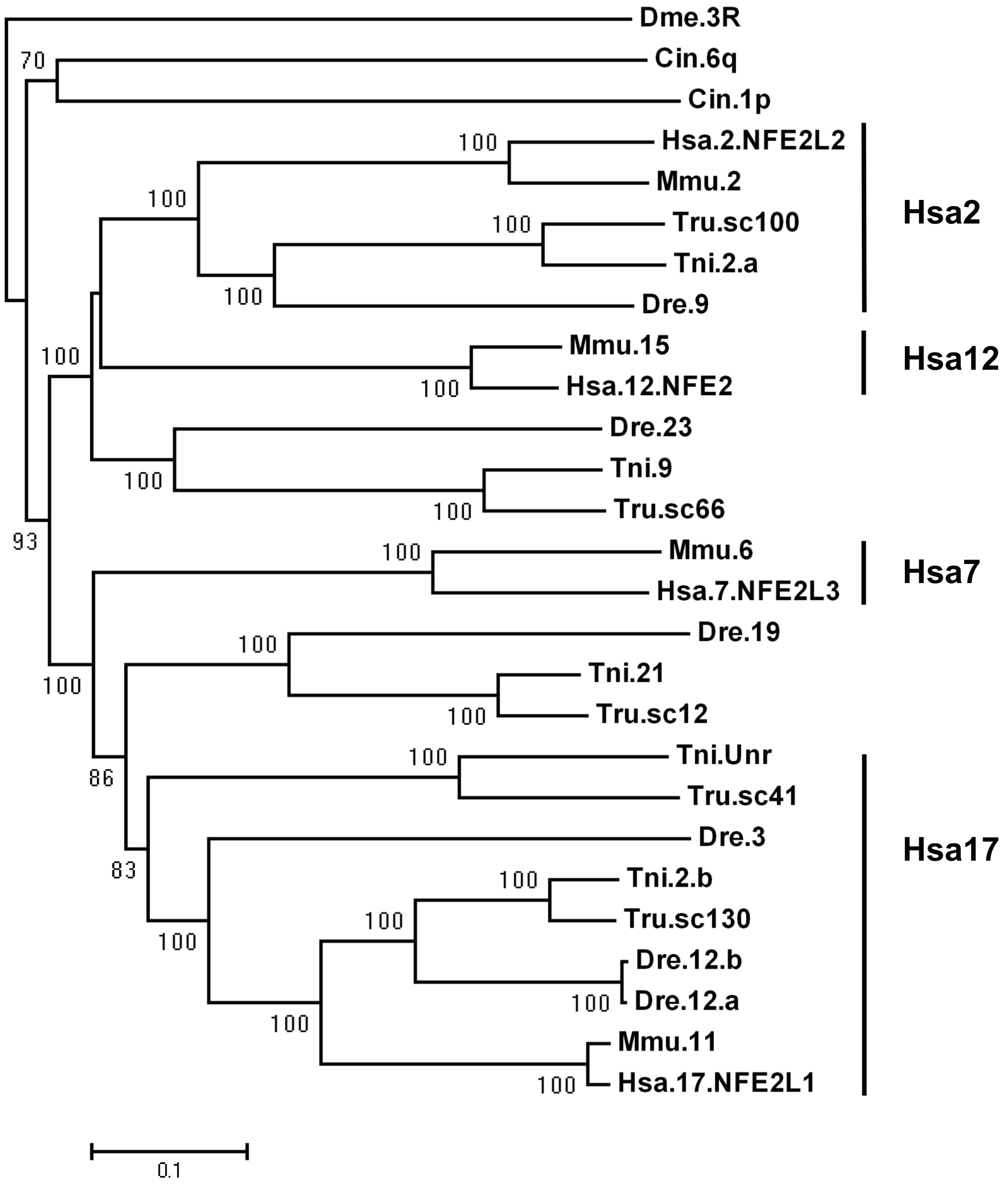

Phylogenetic tree showing the NFE family. Neighbor-joining tree as implemented in ClustalW 1.81 with 1000 bootstrap replicates. Bootstrap percentages shown for each node. Abbreviations as in previous figures.

Supplementary figure 1H (NR1D)

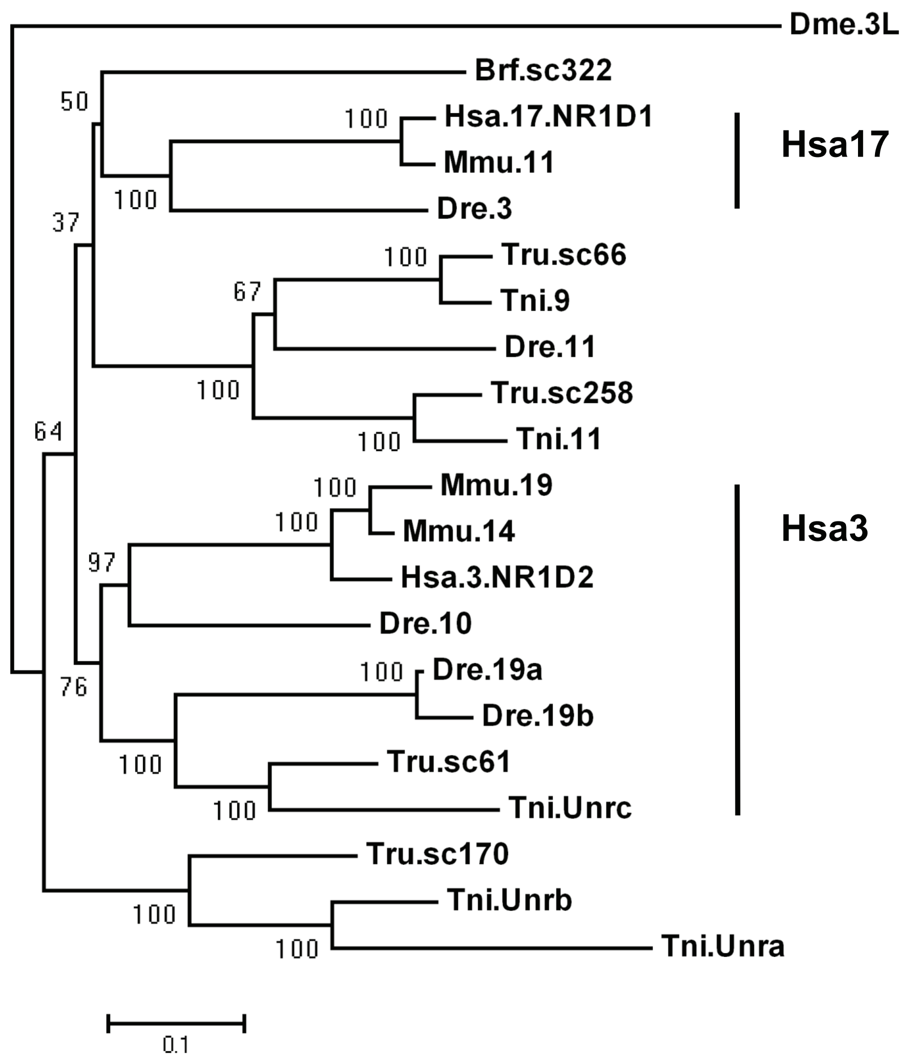

Phylogenetic tree showing the NR1D family. Neighbor-joining tree as implemented in ClustalW 1.81 with 1000 bootstrap replicates. Bootstrap percentages shown for each node. Alignment based on the domains PF00104 (Ligand-binding domain of nuclear hormone receptor) and PF00105 (Zinc finger, C4 type). Abbreviations as in previous figures.

Supplementary figure 1I (OSBPL)

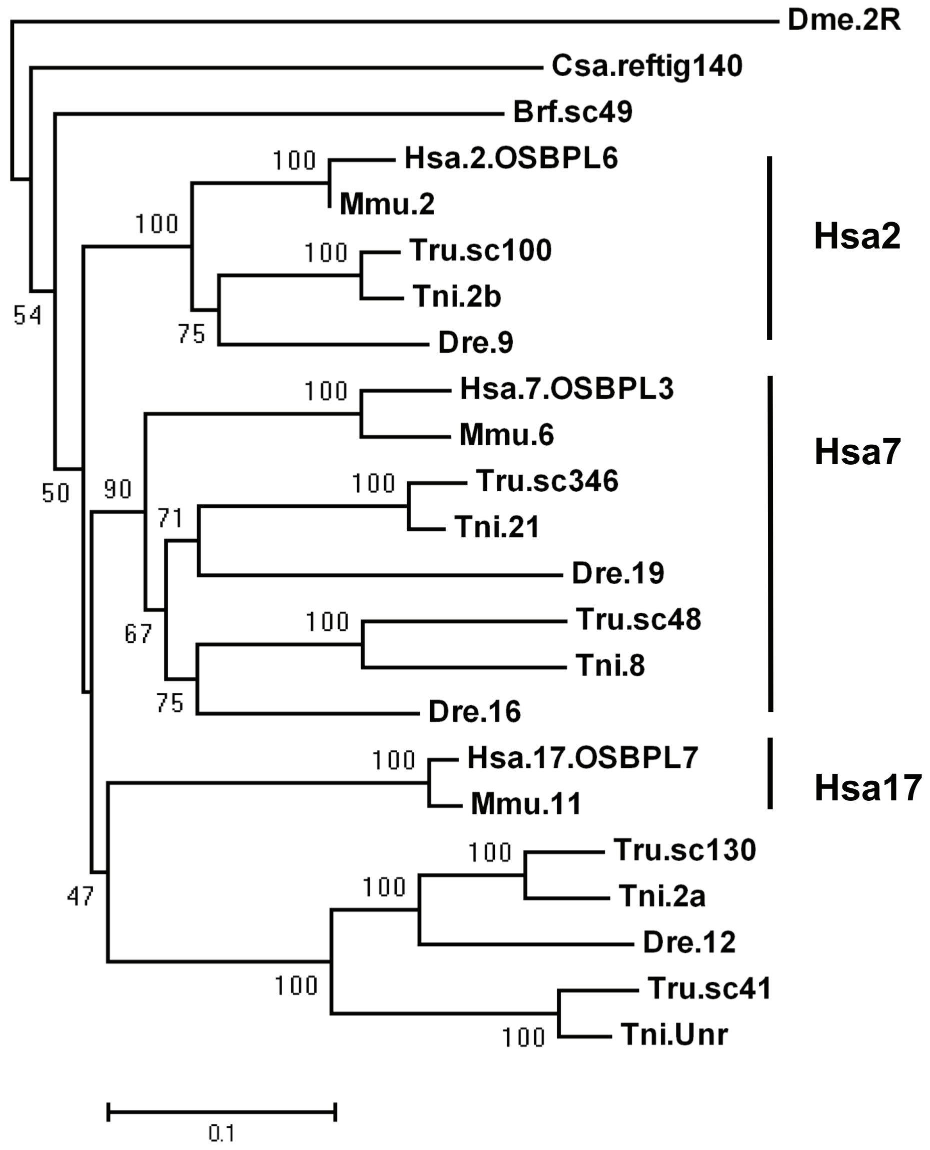

Phylogenetic tree showing the OSBPL family. Neighbor-joining tree as implemented in ClustalW 1.81 with 1000 bootstrap replicates. Bootstrap percentages shown for each node. Alignment based on the domain PF01237 (Oxysterol-binding protein). Abbreviations as in previous figures and Csa is *Ciona savignyi.*

Supplementary figure 1J (RAR)

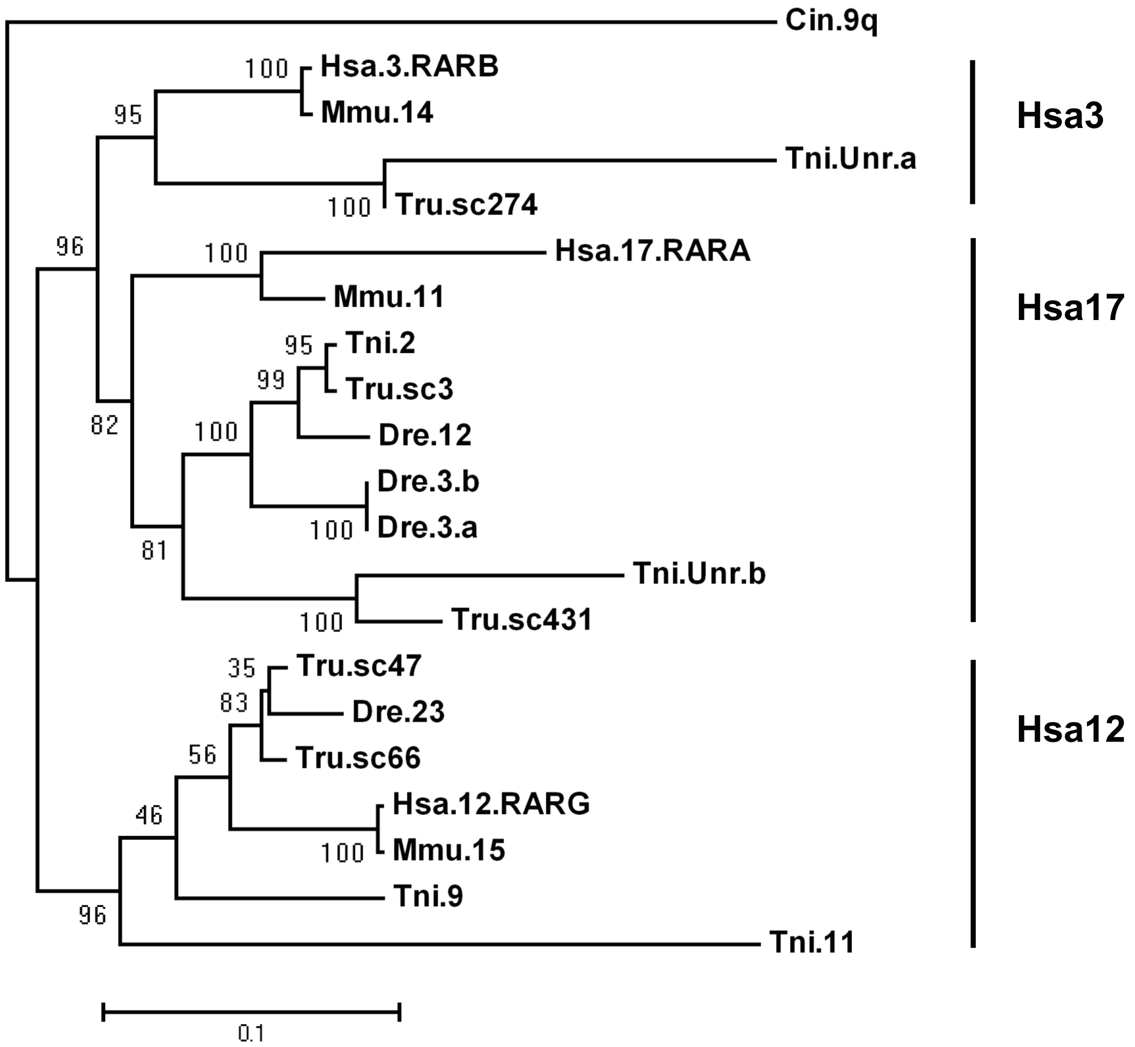

Phylogenetic tree showing the RAR family. Neighbor-joining tree as implemented in ClustalW 1.81 with 1000 bootstrap replicates. Bootstrap percentages shown for each node. Alignment based on the domains PF00104 (Ligand-binding domain of nuclear hormone receptor) and PF00105 (Zinc finger, C4 type). Abbreviations as in previous figures.

Supplementary figure 1K (SLC4A)

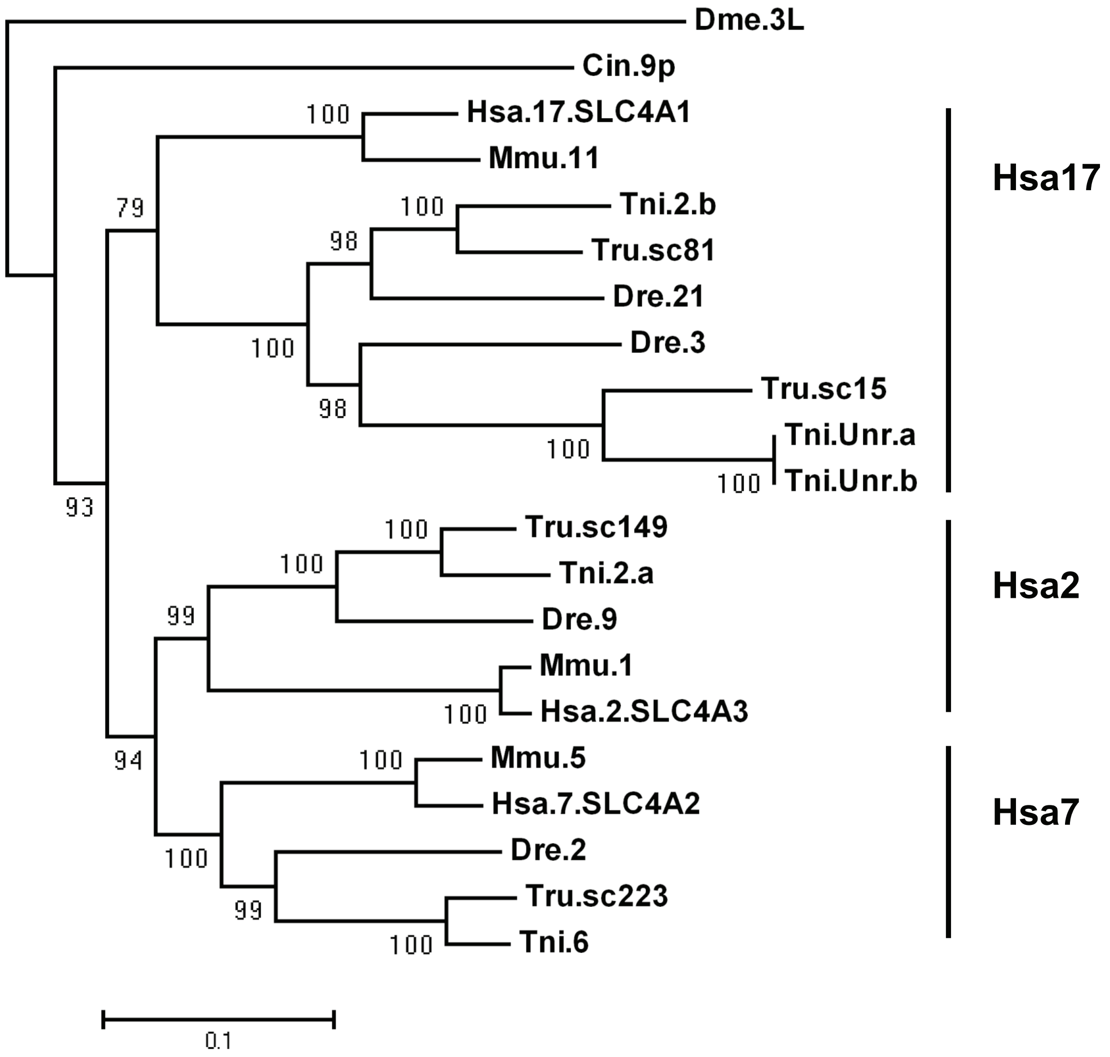

Phylogenetic tree showing the SLC4a family. Neighbor-joining tree as implemented in ClustalW 1.81 with 1000 bootstrap replicates. Bootstrap percentages shown for each node. Alignment based on the domain PF00955 (HCO3- transporter family). Abbreviations as in previous figures.

Supplementary figure 1L (SMARCD)

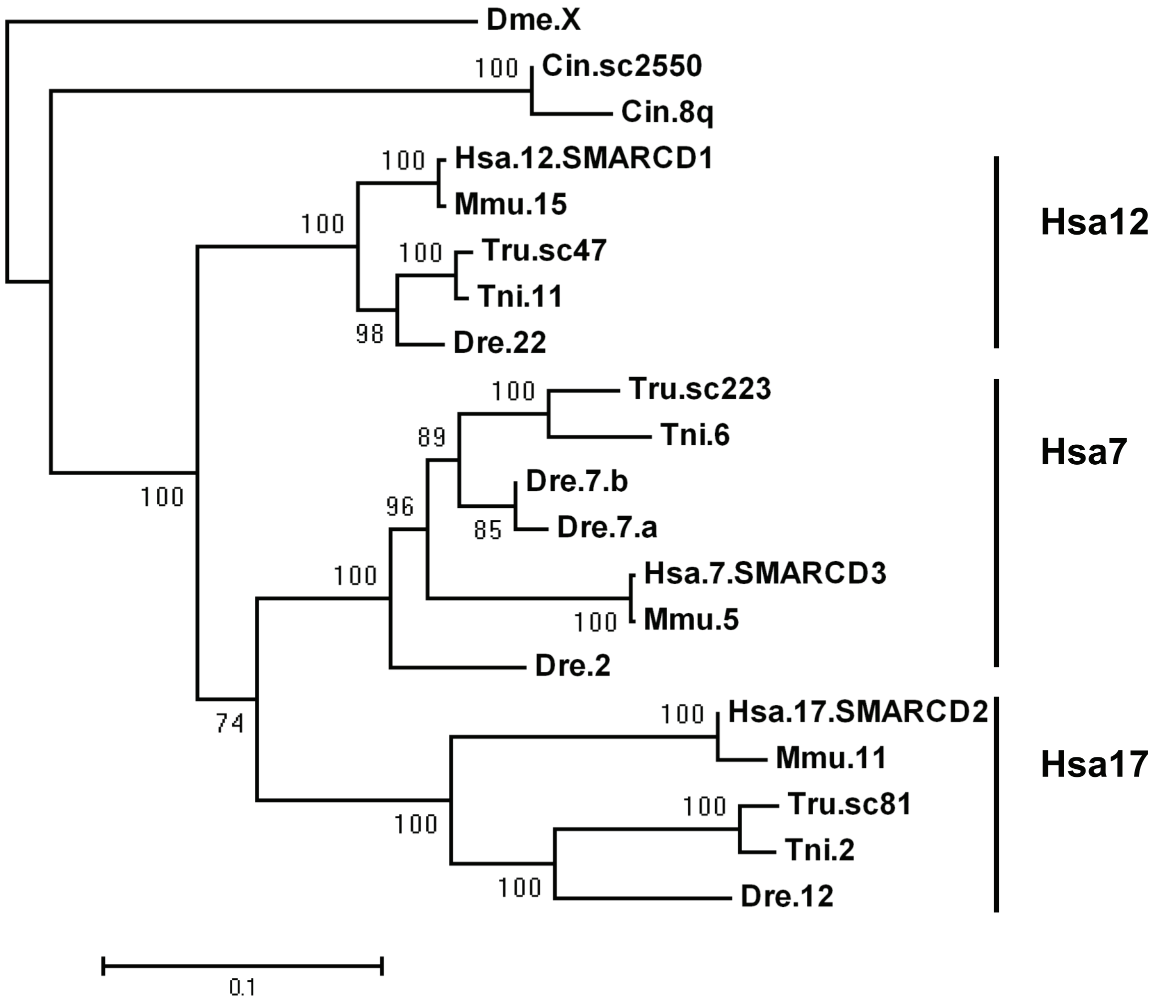

Phylogenetic tree showing the SMARCD family. Neighbor-joining tree as implemented in ClustalW 1.81 with 1000 bootstrap replicates. Bootstrap percentages shown for each node. Abbreviations as in previous figures.

Supplementary figure 1M (THR)

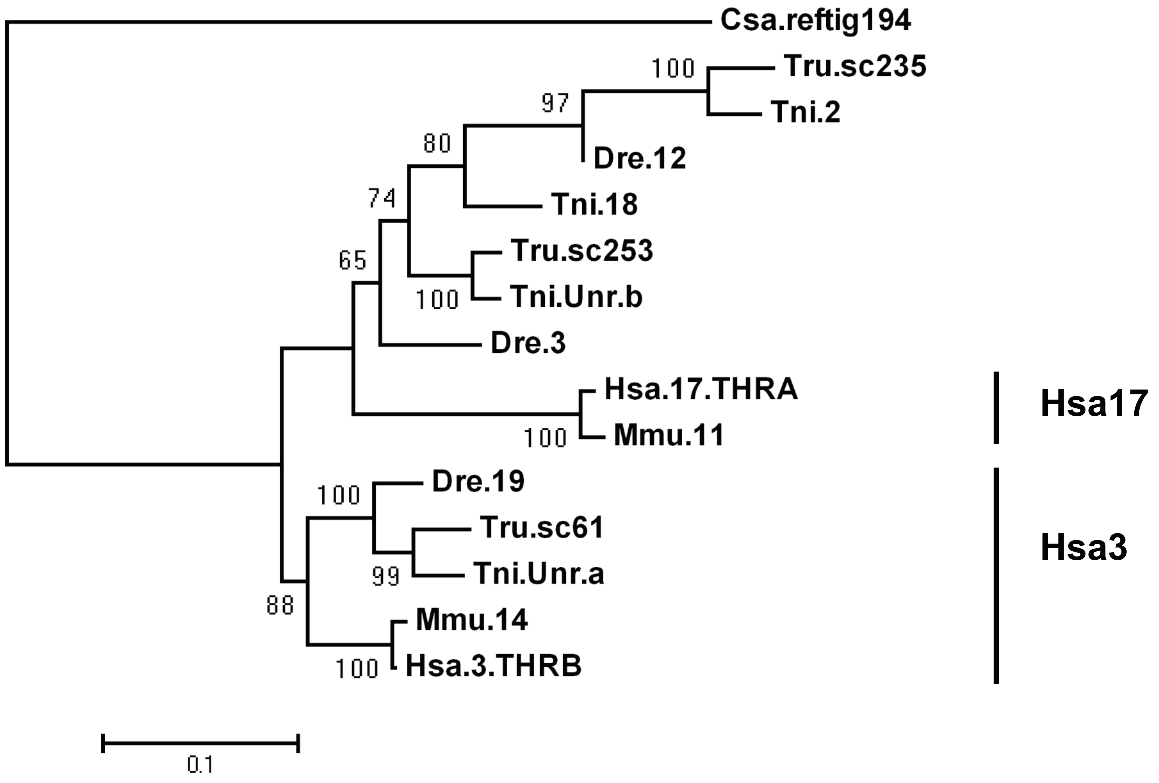

Phylogenetic tree showing the THR family. Neighbor-joining tree as implemented in ClustalW 1.81 with 1000 bootstrap replicates. Bootstrap percentages shown for each node. Alignment based on the domains PF00104 (Ligand-binding domain of nuclear hormone receptor) and PF00105 (Zinc finger, C4 type). Abbreviations as in previous figures.

Supplementary figure 1N (UPP)

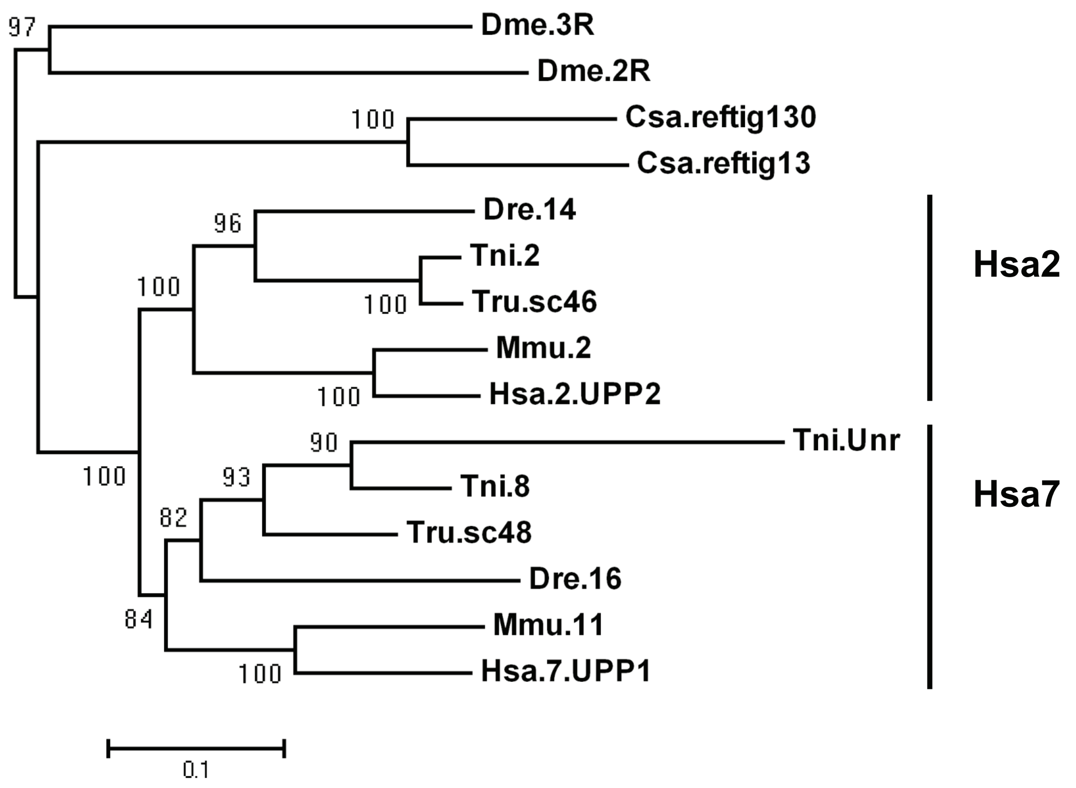

Phylogenetic tree showing the UPP family. Neighbor-joining tree as implemented in ClustalW 1.81 with 1000 bootstrap replicates. Bootstrap percentages shown for each node. Abbreviations as in previous figures.
