# Supplementary material for: Phylogenetic and chromosomal analyses of multiple gene families syntenic with vertebrate Hox clusters
Source: BMC Evol Biol. 2008 Sep 19;8:254. doi: 10.1186/1471-2148-8-254 (PMC2566581; doi:10.1186/1471-2148-8-254)
Supplement: Additional file 2 — Quartet puzzling trees for the 14 gene families analyzed. [file 1471-2148-8-254-S2.pdf]

Supplementary figure 2A (AOC)

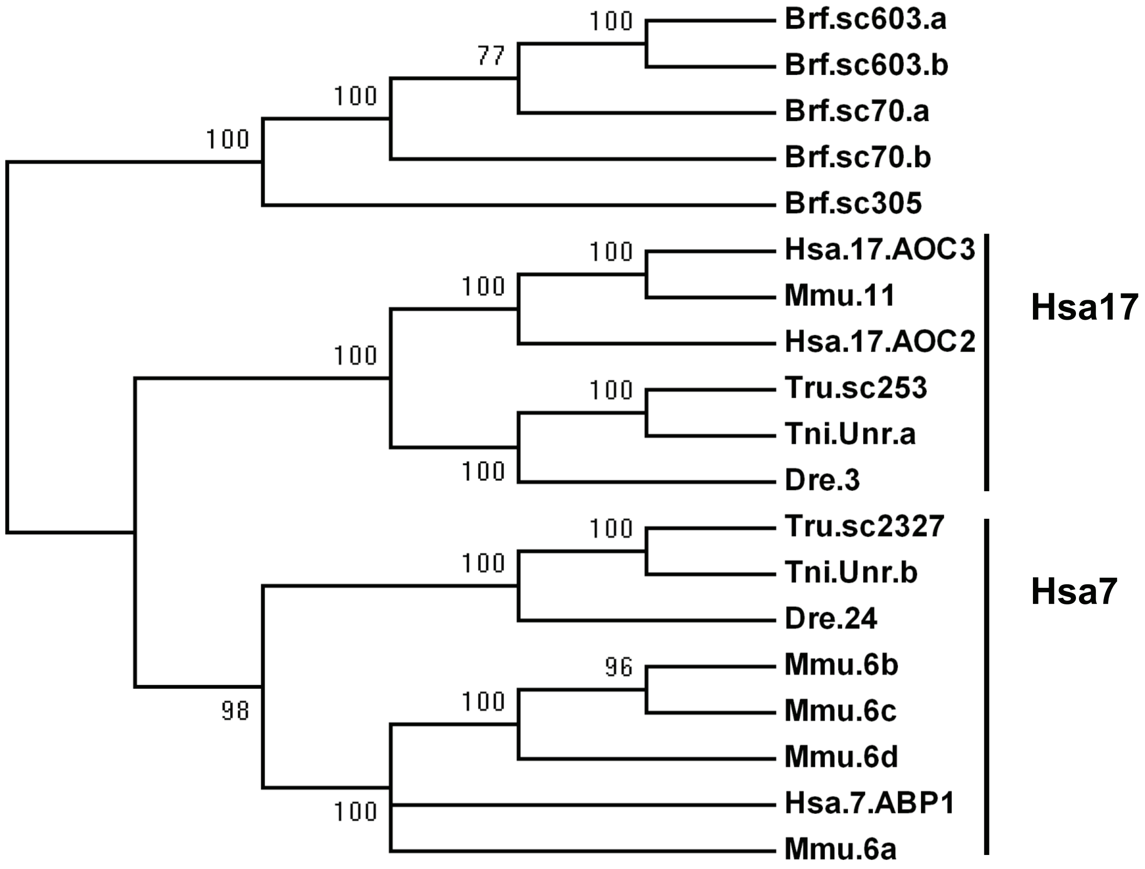

Phylogenetic tree showing the AOC family. Quartet-puzzling tree as implemented in Treepuzzle 5.2. Quartet-puzzling support values shown for each node. Hsa is human (*Homo sapiens*), Mmu: Mouse (*Mus musculus*), Dre: zebrafish (*Danio rerio*), Tru: fugu (*Takifugu rubripes*),Tni: tetraodon (*Tetraodon nigroviridis)* and Brf: amphioxus (*Branchiostoma floridae*).

The tree was constructed using the following settings:

Parameter estimation: accurate (slow)

Parameter estimation uses: quartet sampling (for substitution process) + NJ tree (for rate variation)

Model of substitution: JTT

Amino acid frequencies estimated from data set

Model of rate heterogeneity: Gamma distributed rates

Gamma distribution parameter alpha estimated from data set

Number of Gamma rate categories: 8

Number of puzzling steps: 1000

Supplementary figure 2B (DLX)

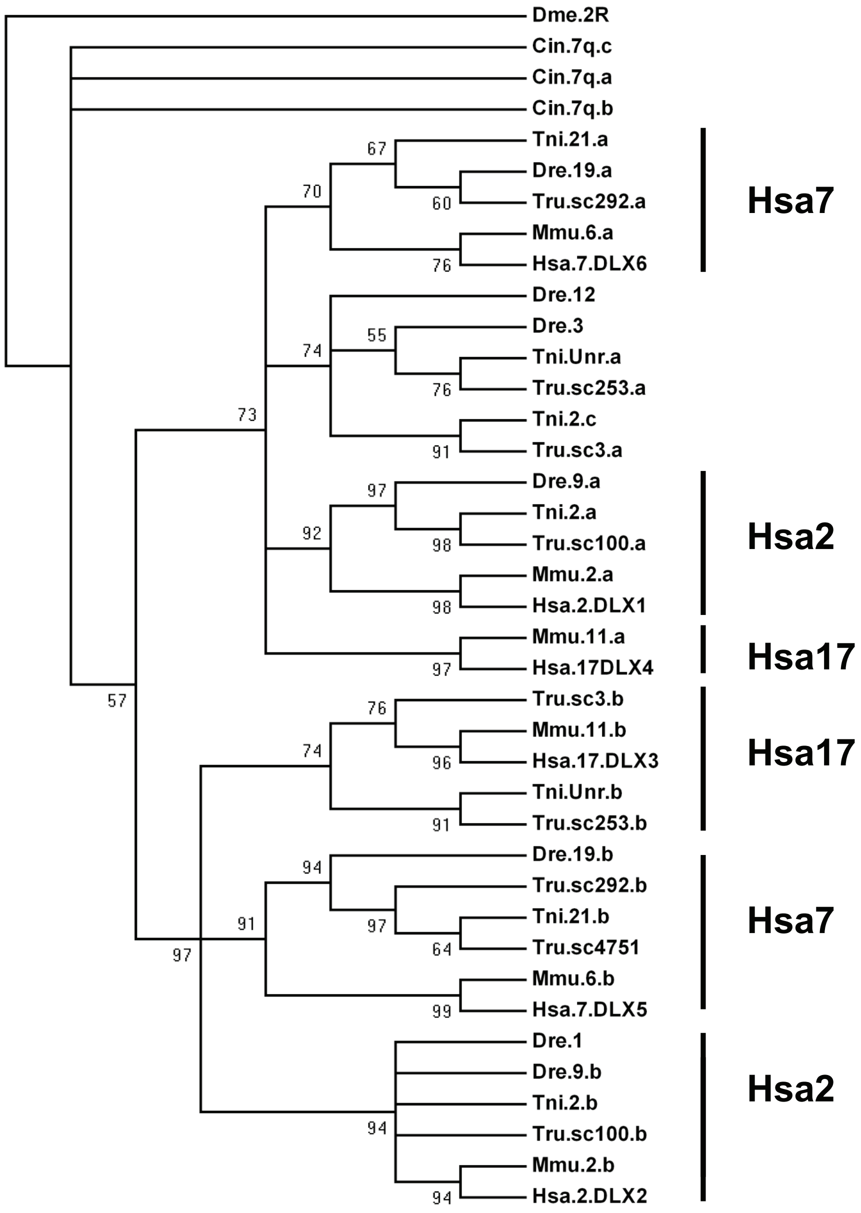

Phylogenetic tree showing the DLX family. Quartet-puzzling tree as implemented in Treepuzzle 5.2. Quartet-puzzling support values shown for each node. Sequences without the homeodomain (pfam ID: PF00046) were removed from the alignment. Abbreviations as in figure 2A and Cin is *Ciona intestinalis* and Dme: *Drosophila melanogaster*.

The tree was constructed using the following settings:

Parameter estimation: accurate (slow)

Parameter estimation uses: quartet sampling (for substitution process) + NJ tree (for rate variation)

Model of substitution: Dayhoff

Amino acid frequencies estimated from data set

Model of rate heterogeneity: Gamma distributed rates

Gamma distribution parameter alpha estimated from data set.

Number of Gamma rate categories: 8

Number of puzzling steps: 10000

Supplementary figure 2C (G6PC)

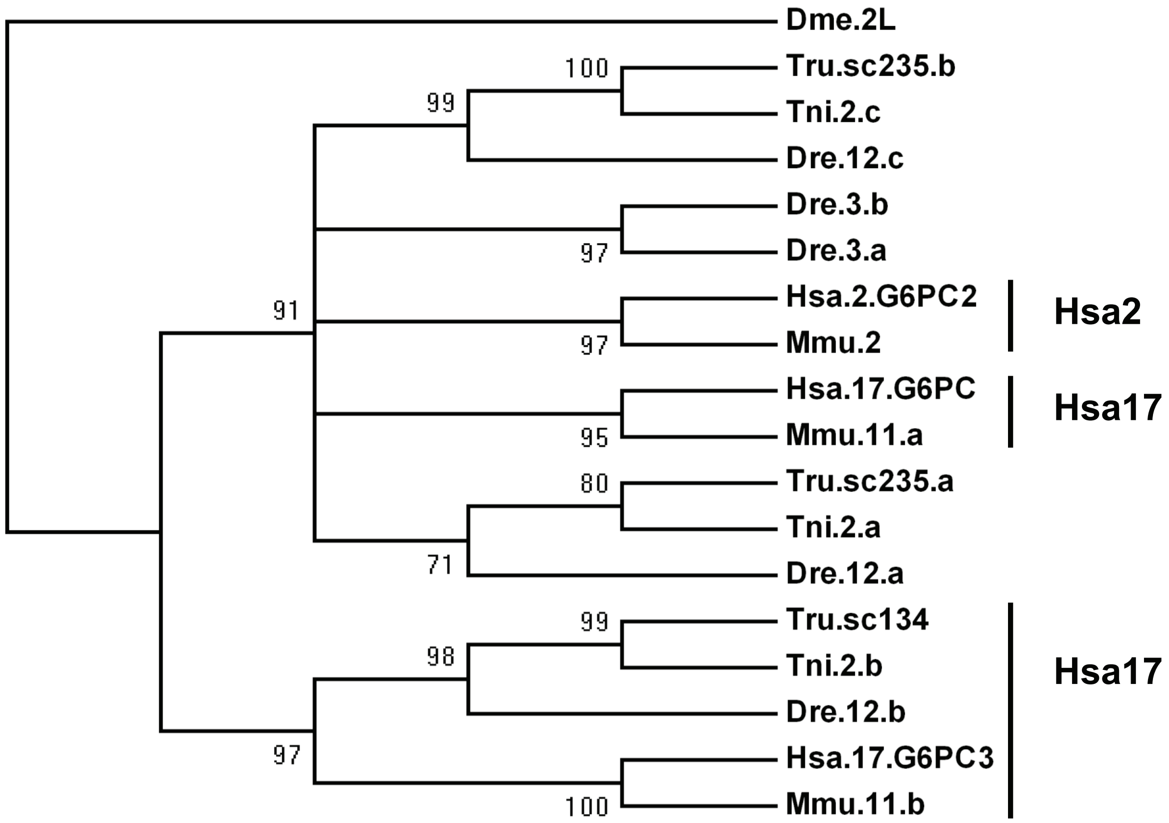

Phylogenetic tree showing the G6PC family. Quartet-puzzling tree as implemented in Treepuzzle 5.2. Quartet-puzzling support values shown for each node. Abbreviations as in previous figures.

The tree was constructed using the following settings:
Parameter estimation: accurate (slow)
Parameter estimation uses: quartet sampling (for substitution process) + NJ tree (for rate variation)
Model of substitution: JTT
Amino acid frequencies estimated from data set
Model of rate heterogeneity: Gamma distributed rates
Gamma distribution parameter alpha estimated from data set
Number of Gamma rate categories: 8
Number of puzzling steps: 1000

Supplementary figure 2D (IGFBP)

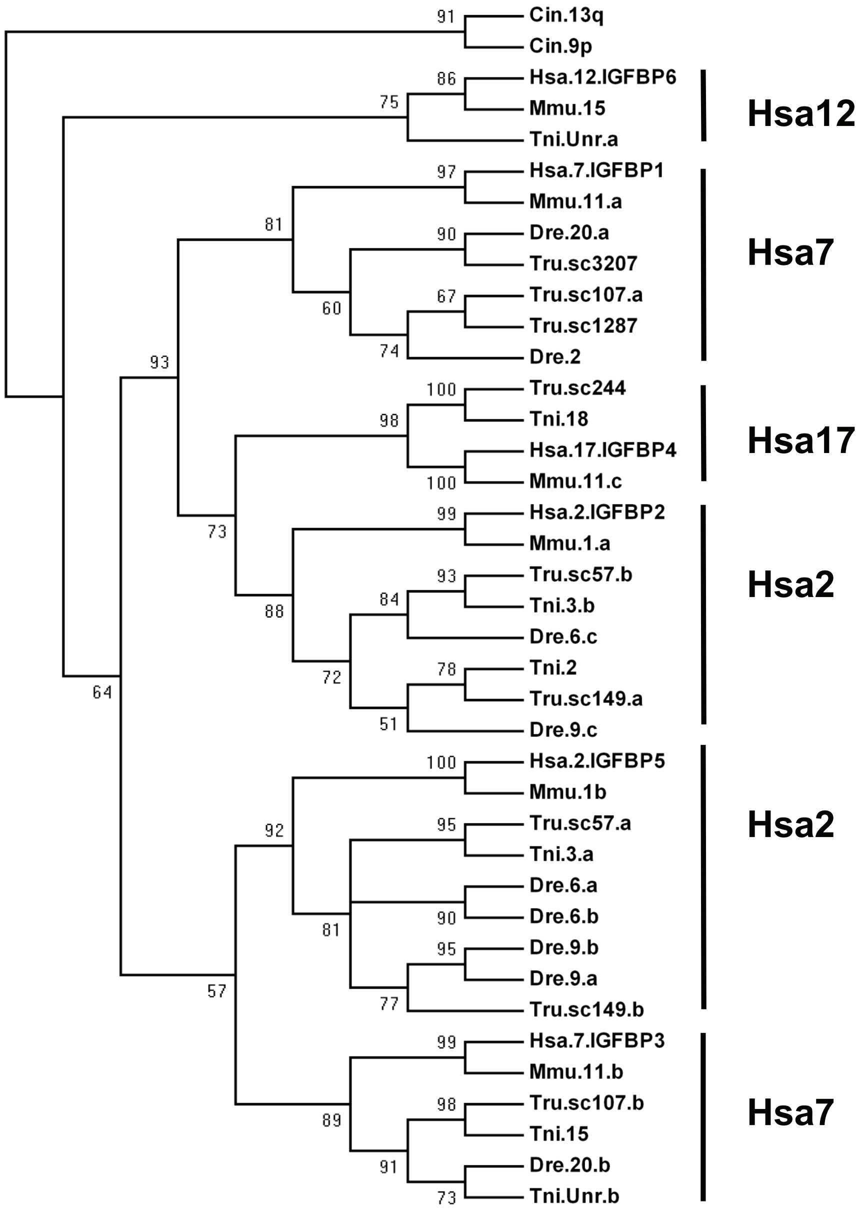

Phylogenetic tree showing the IGFBP family. Quartet-puzzling tree as implemented in Treepuzzle 5.2. Quartet-puzzling support values shown for each node. Sequences without the domains PF00219 (Insulin-like growth factor binding protein) and PF00086 (Thyroglobulin type-1 repeat) were removed from the alignment. Abbreviations as in previous figures. Note that the tree contains two subfamilies relevant for this paralogon.

The tree was constructed using the following settings:
Parameter estimation: accurate (slow)
Parameter estimation uses: quartet sampling (for substitution process) + NJ tree (for rate variation)
Model of substitution: Dayhoff
Amino acid frequencies estimated from data set
Model of rate heterogeneity: Gamma distributed rates
Gamma distribution parameter alpha estimated from data set
Number of Gamma rate categories: 8
Number of puzzling steps: 10000

Supplementary figure 2E (IGF2BP)

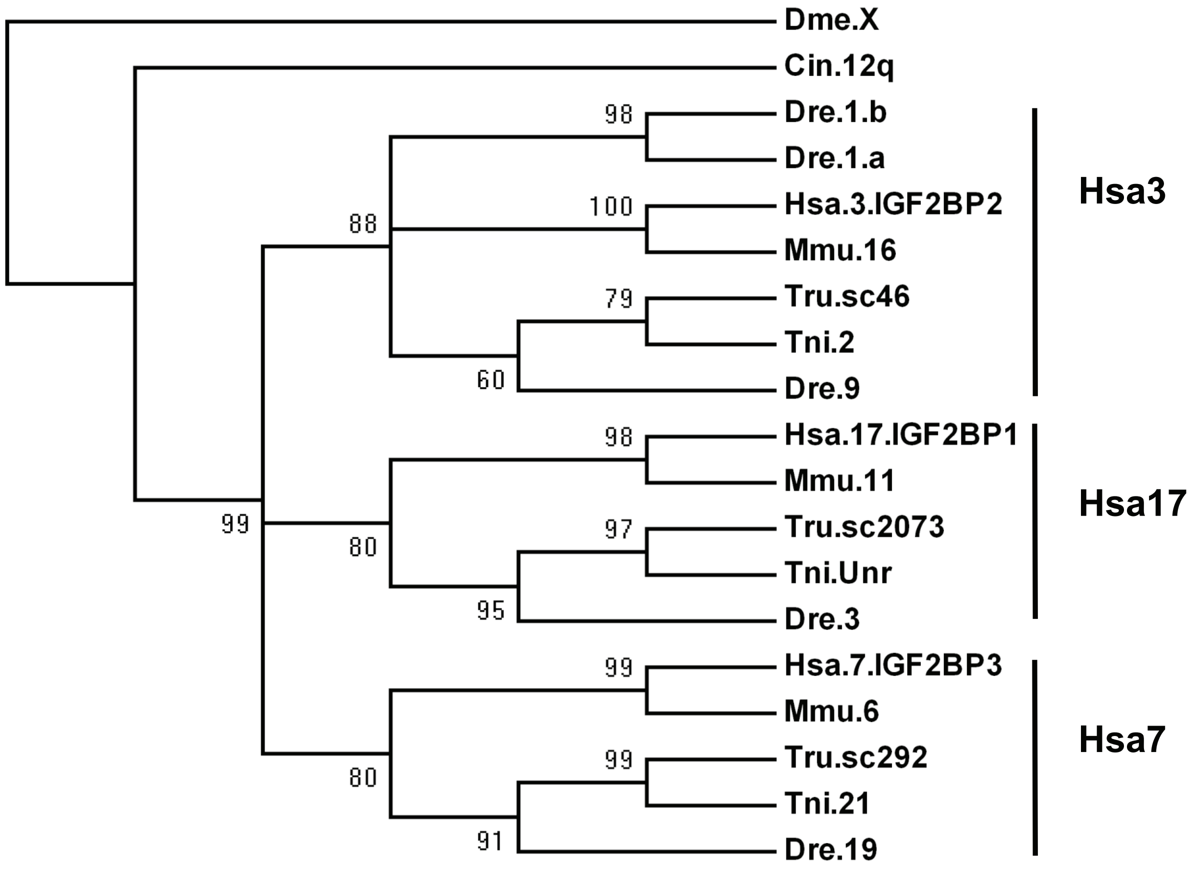

Phylogenetic tree showing the IGF2BP family. Quartet-puzzling tree as implemented in Treepuzzle 5.2. Quartet-puzzling support values shown for each node. Abbreviations as in previous figures.

The tree was constructed using the following settings:
Parameter estimation: accurate (slow)
Parameter estimation uses: quartet sampling (for substitution process) + NJ tree (for rate variation)
Model of substitution: JTT
Amino acid frequencies estimated from data set
Model of rate heterogeneity: Gamma distributed rates
Gamma distribution parameter alpha estimated from data set
Number of Gamma rate categories: 8
Number of puzzling steps: 1000

Supplementary figure 2F (MPP)

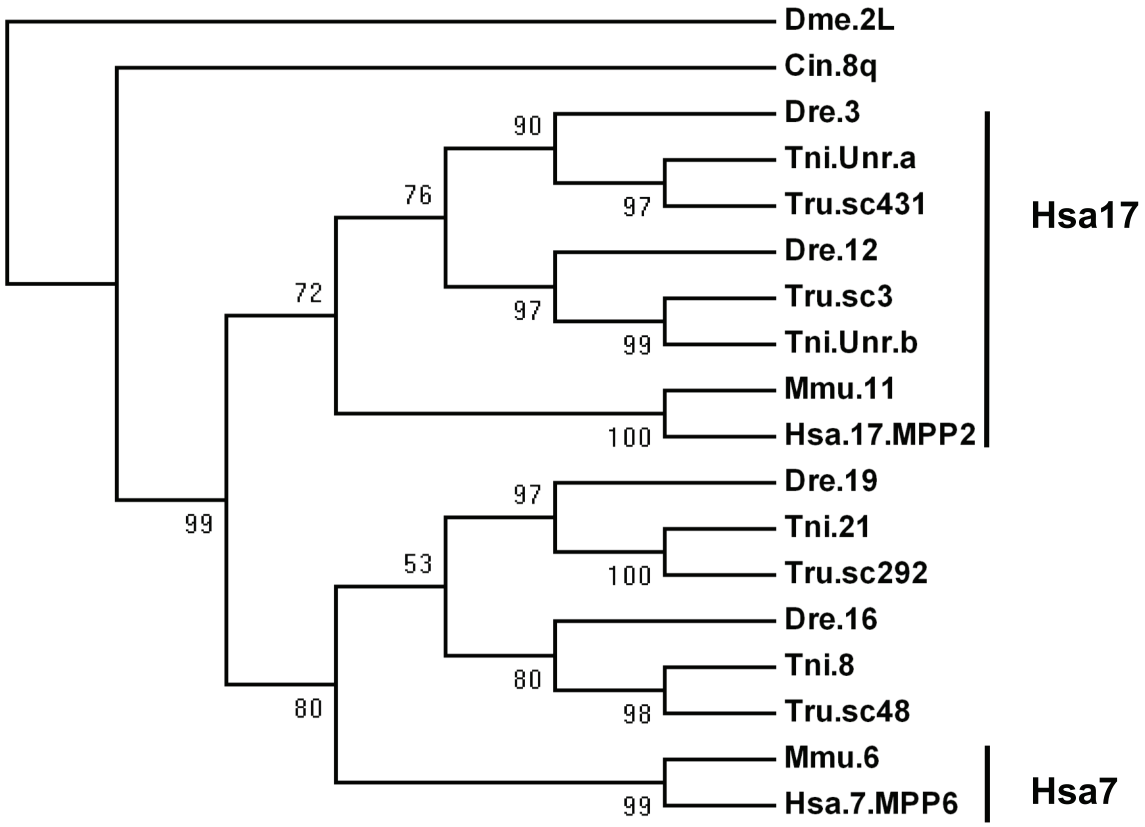

Phylogenetic tree showing the MPP family. Quartet-puzzling tree as implemented in Treepuzzle 5.2. Quartet-puzzling support values shown for each node. Alignment based on the domains PF00595 (PDZ domain), PF00018 (SH3 domain) and PF00625 (Guanylate kinase). Sequences without the domain PF00625 were removed from the alignment. Abbreviations as in previous figures.

The tree was constructed using the following settings:

Parameter estimation: accurate (slow)

Parameter estimation uses: quartet sampling (for substitution process) + NJ tree (for rate variation)

Model of substitution: JTT

Amino acid frequencies estimated from data set

Model of rate heterogeneity: Gamma distributed rates

Gamma distribution parameter alpha estimated from data set

Number of Gamma rate categories: 8

Number of puzzling steps: 1000

Supplementary figure 2G (NFE)

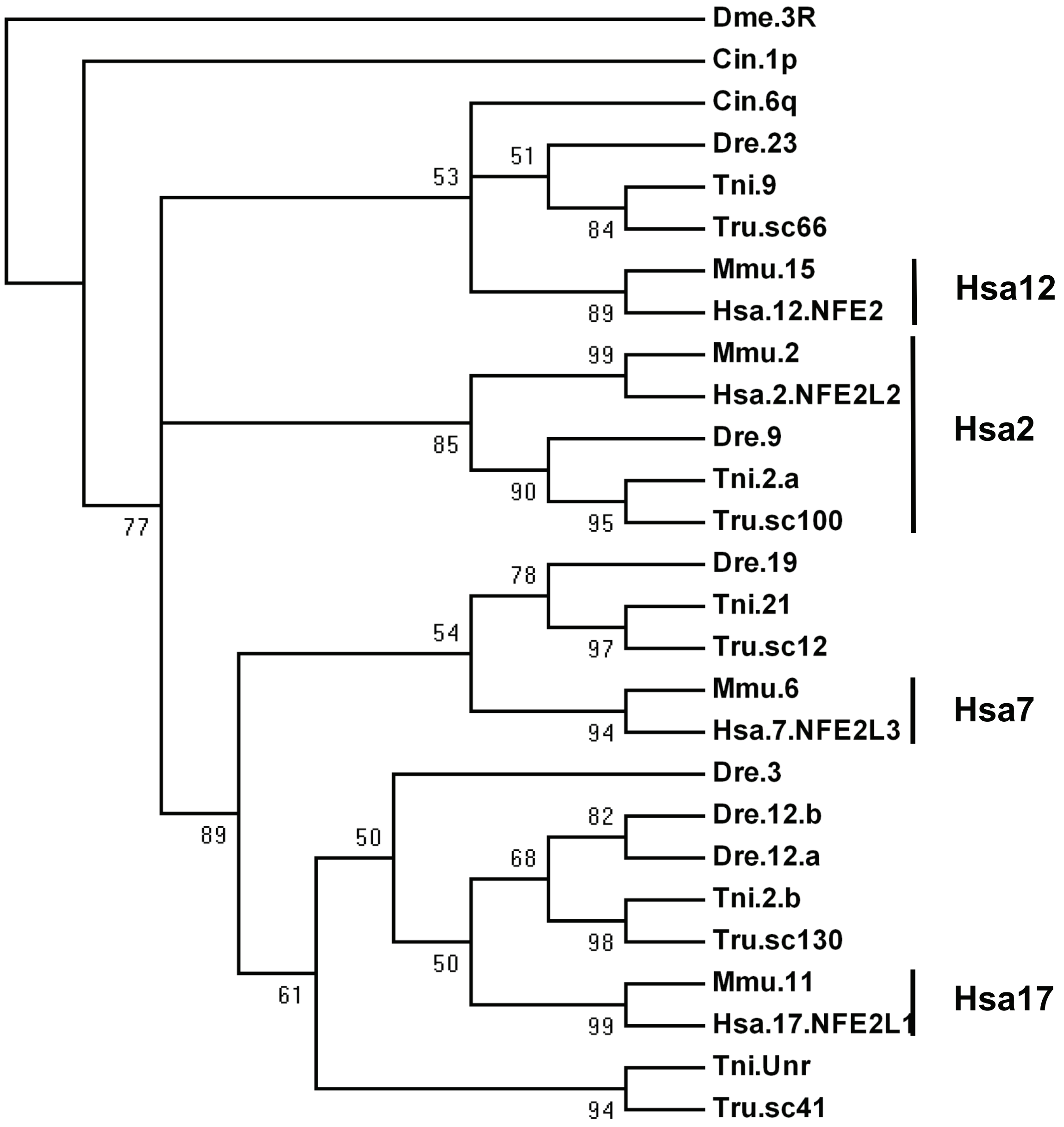

Phylogenetic tree showing the NFE family. Quartet-puzzling tree as implemented in Treepuzzle 5.2. Quartet-puzzling support values shown for each node. Abbreviations as in previous figures.

The tree was constructed using the following settings:

Parameter estimation: accurate (slow)

Parameter estimation uses: quartet sampling (for substitution process) + NJ tree (for rate variation)

Model of substitution: VT

Amino acid frequencies estimated from data set

Model of rate heterogeneity: Gamma distributed rates

Gamma distribution parameter alpha estimated from data set

Number of Gamma rate categories: 8

Number of puzzling steps: 10000

Supplementary figure 2H (NR1D)

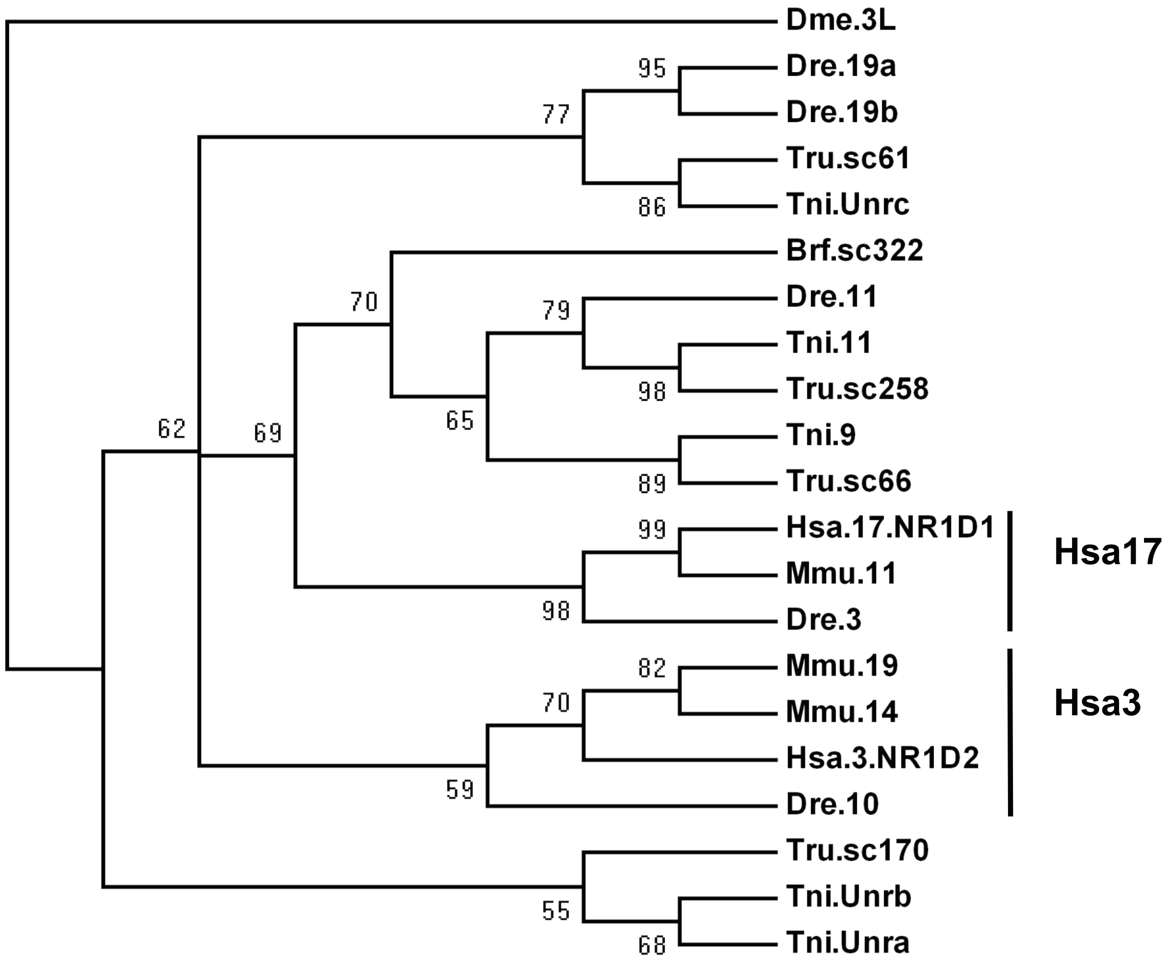

Phylogenetic tree showing the NR1D family. Quartet-puzzling tree as implemented in Treepuzzle 5.2. Quartet-puzzling support values shown for each node. Alignment based on the domains PF00104 (Ligand-binding domain of nuclear hormone receptor) and PF00105 (Zinc finger, C4 type). Abbreviations as in previous figures.

The tree was constructed using the following settings:

Parameter estimation: accurate (slow)

Parameter estimation uses: quartet sampling (for substitution process) + NJ tree (for rate variation)

Model of substitution: JTT

Amino acid frequencies estimated from data set

Model of rate heterogeneity: Gamma distributed rates

Gamma distribution parameter alpha estimated from data set

Number of Gamma rate categories: 8

Number of puzzling steps: 1000

Supplementary figure 2I (OSBPL)

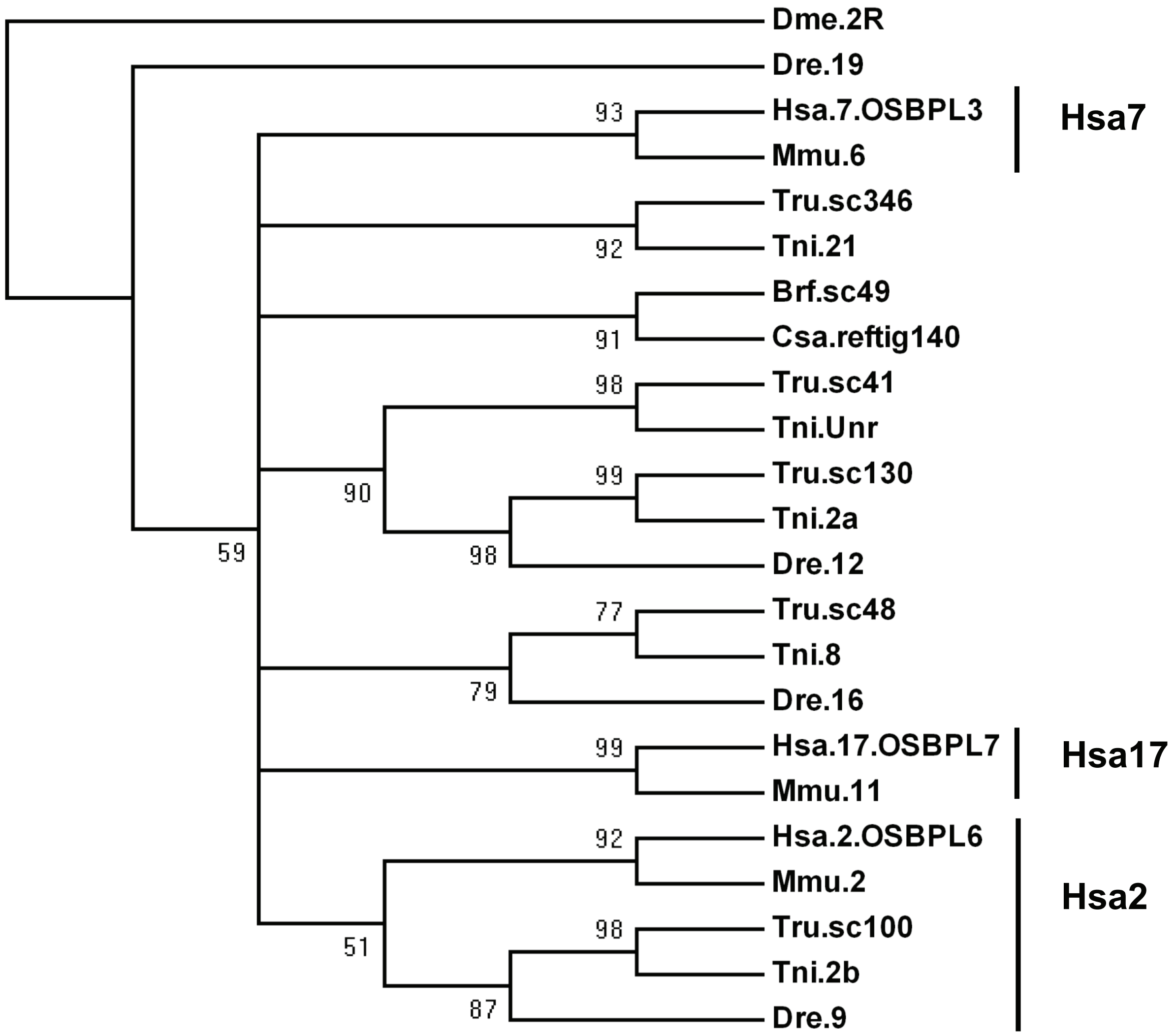

Phylogenetic tree showing the OSBPL family. Quartet-puzzling tree as implemented in Treepuzzle 5.2. Quartet-puzzling support values shown for each node. Alignment based on the domain PF01237 (Oxysterol-binding protein). Abbreviations as in previous figures and Csa is *Ciona savignyi.*
The tree was constructed using the following settings:
Parameter estimation: accurate (slow)
Parameter estimation uses: quartet sampling (for substitution process) + NJ tree (for rate variation)
Model of substitution: JTT
Amino acid frequencies estimated from data set
Model of rate heterogeneity: Gamma distributed rates
Gamma distribution parameter alpha estimated from data set
Number of Gamma rate categories: 8
Number of puzzling steps: 1000

Supplementary figure 2J (RAR)

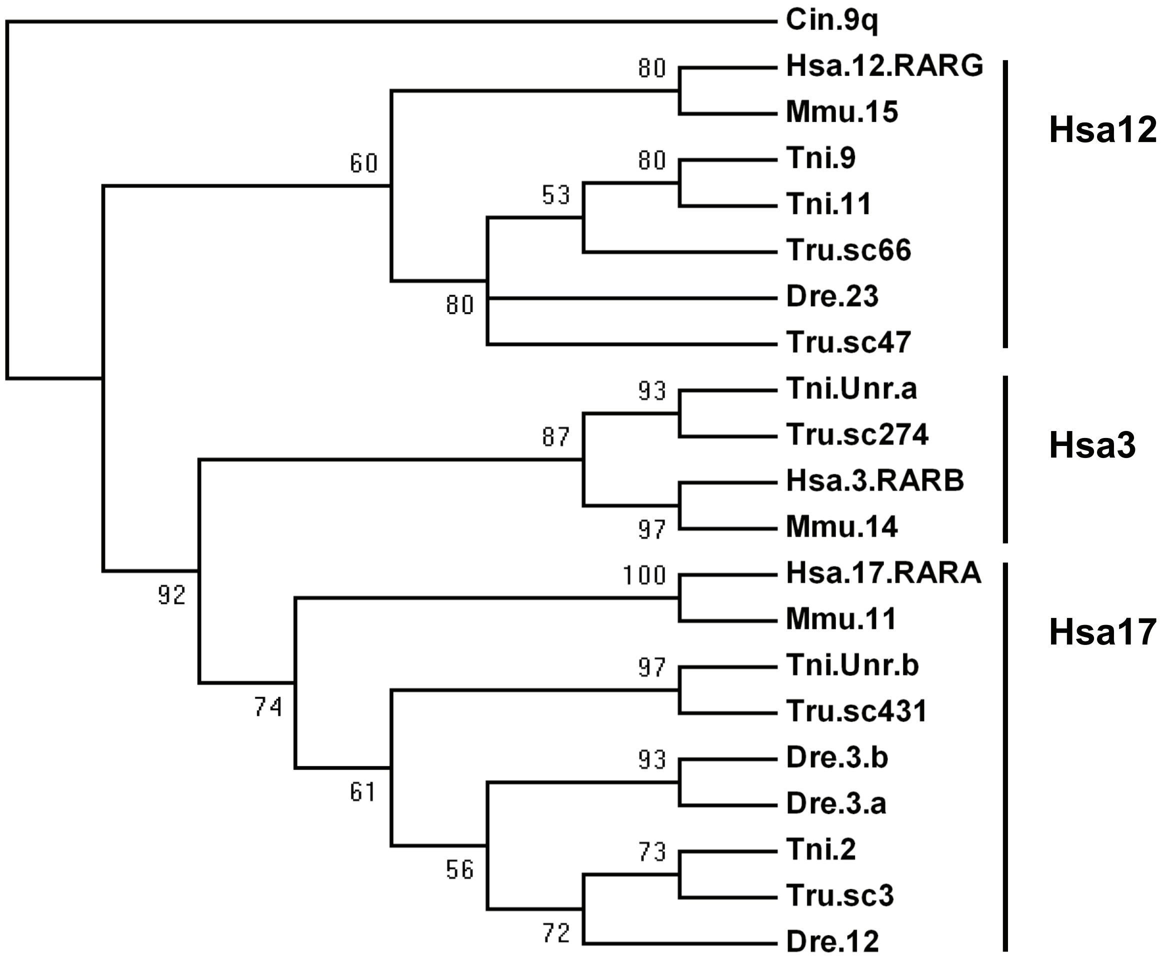

Phylogenetic tree showing the RAR family. Quartet-puzzling tree as implemented in Treepuzzle 5.2. Quartet-puzzling support values shown for each node. Alignment based on the domains PF00104 (Ligand-binding domain of nuclear hormone receptor) and PF00105 (Zinc finger, C4 type). Abbreviations as in previous figures.

The tree was constructed using the following settings:

Parameter estimation: accurate (slow)

Parameter estimation uses: quartet sampling (for substitution process) + NJ tree (for rate variation)

Model of substitution: JTT

Amino acid frequencies estimated from data set

Model of rate heterogeneity: Gamma distributed rates

Gamma distribution parameter alpha estimated from data set

Number of Gamma rate categories: 8

Number of puzzling steps: 1000

Supplementary figure 2K (SLC4A)

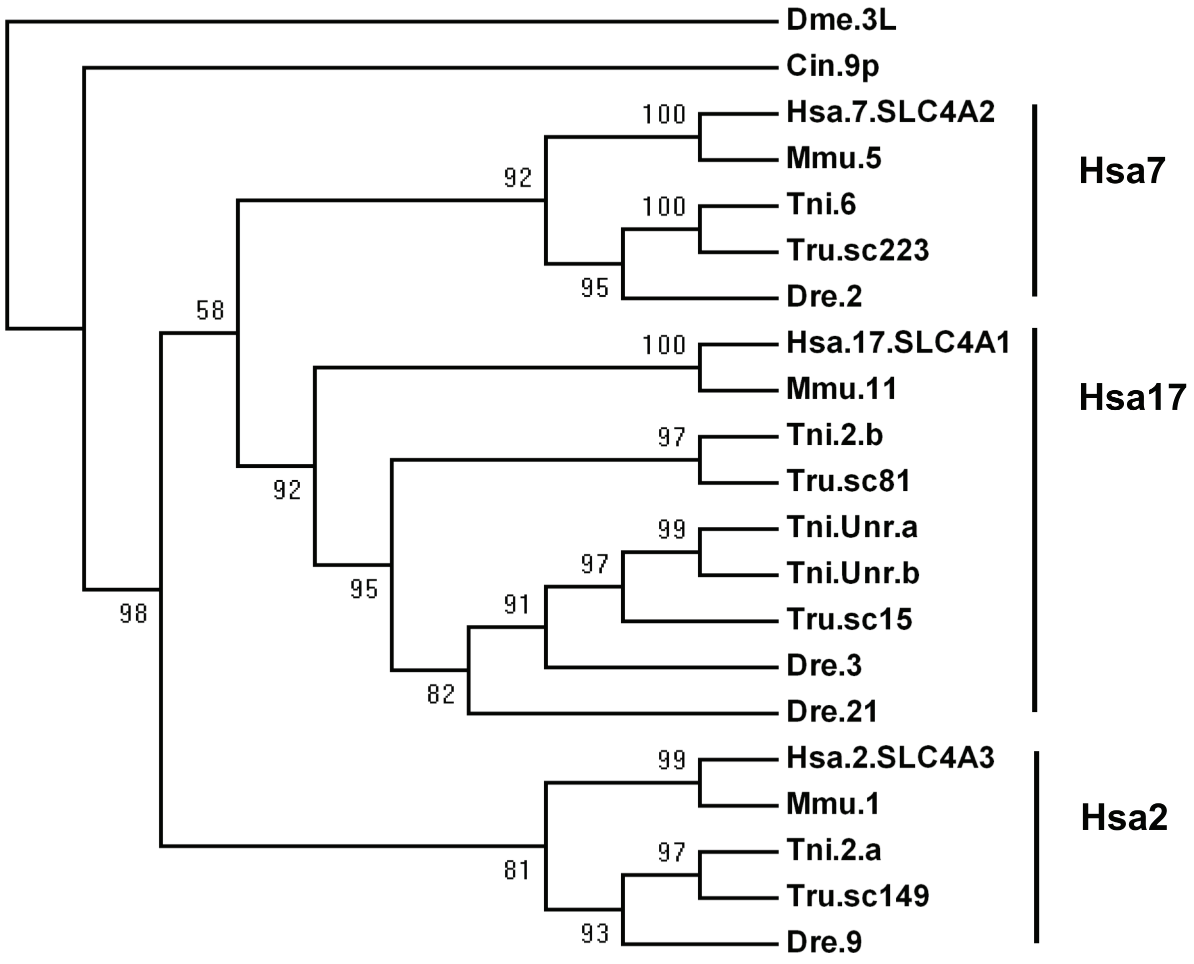

Phylogenetic tree showing the SLC4A family. Quartet-puzzling tree as implemented in Treepuzzle 5.2. Quartet-puzzling support values shown for each node. Alignment based on the domain PF00955 (HCO3- transporter family). Abbreviations as in previous figures. The tree was constructed using the following settings:

Parameter estimation: accurate (slow)

Parameter estimation uses: quartet sampling (for substitution process) + NJ tree (for rate variation)

Model of substitution: VT

Amino acid frequencies estimated from data set

Model of rate heterogeneity: Gamma distributed rates

Gamma distribution parameter alpha estimated from data set

Number of Gamma rate categories: 8

Number of puzzling steps: 1000

Supplementary figure 2L (SMARCD)

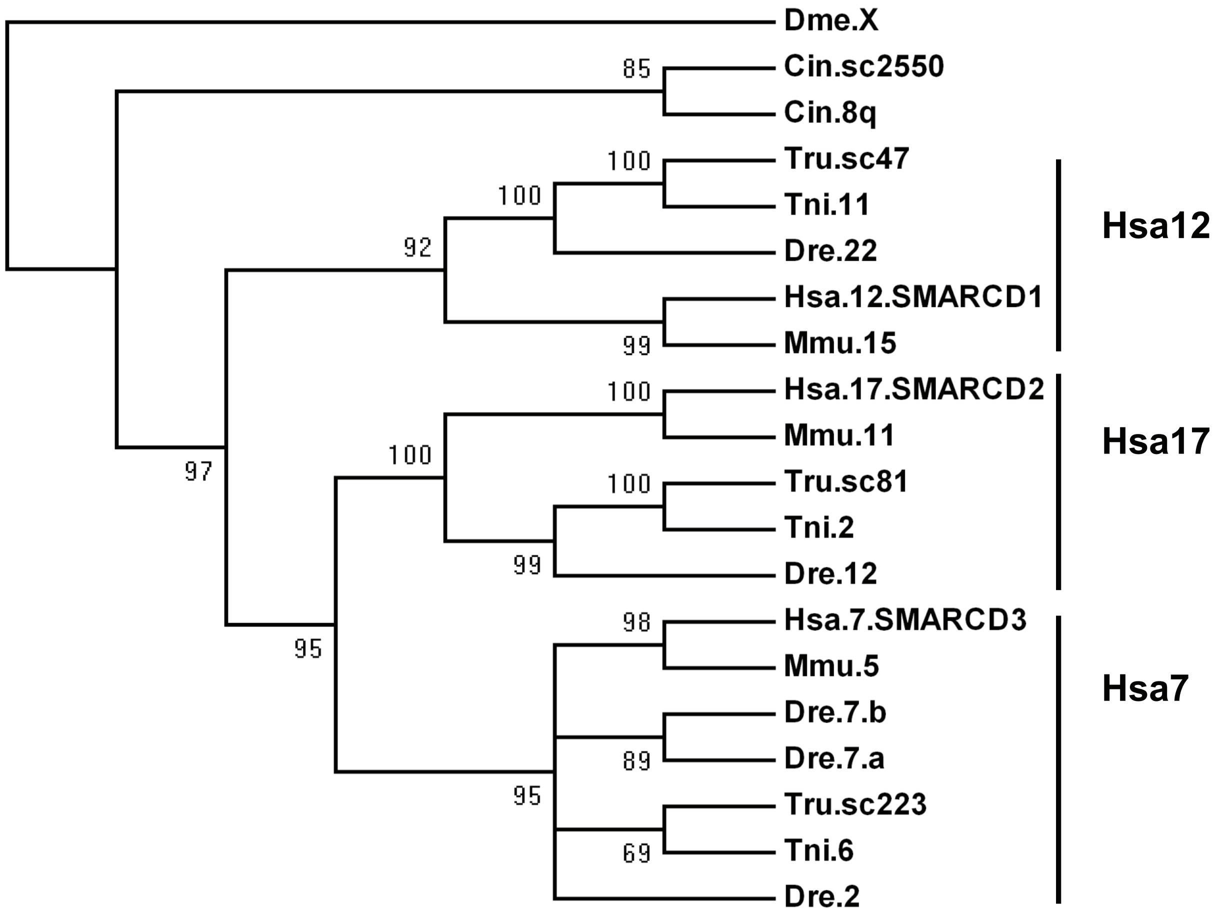

Phylogenetic tree showing the SMARCD family. Quartet-puzzling tree as implemented in Treepuzzle 5.2. Quartet-puzzling support values shown for each node. Abbreviations as in previous figures.

The tree was constructed using the following settings:
Parameter estimation: accurate (slow)
Parameter estimation uses: quartet sampling (for substitution process) + NJ tree (for rate variation)
Model of substitution: JTT
Amino acid frequencies estimated from data set
Model of rate heterogeneity: Gamma distributed rates
Gamma distribution parameter alpha estimated from data set
Number of Gamma rate categories: 8
Number of puzzling steps: 1000

Supplementary figure 2M (THR)

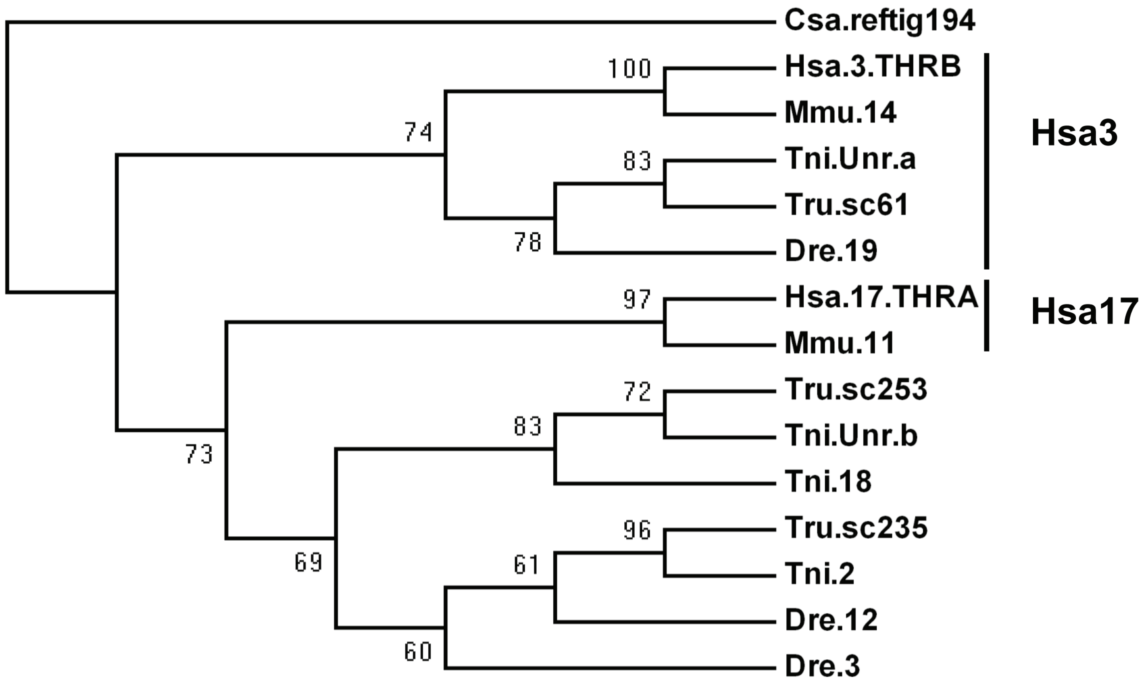

Phylogenetic tree showing the THR family. Quartet-puzzling tree as implemented in Treepuzzle 5.2. Quartet-puzzling support values shown for each node. Alignment based on the domains PF00104 (Ligand-binding domain of nuclear hormone receptor) and PF00105 (Zinc finger, C4 type). Abbreviations as in previous figures and Csa is *Ciona savignyi.*
The tree was constructed using the following settings:
Parameter estimation: accurate (slow)
Parameter estimation uses: quartet sampling (for substitution process) + NJ tree (for rate variation)
Model of substitution: VT
Amino acid frequencies estimated from data set
Model of rate heterogeneity: Gamma distributed rates
Gamma distribution parameter alpha estimated from data set
Number of Gamma rate categories: 8
Number of puzzling steps: 1000

Supplementary figure 2N (UPP)

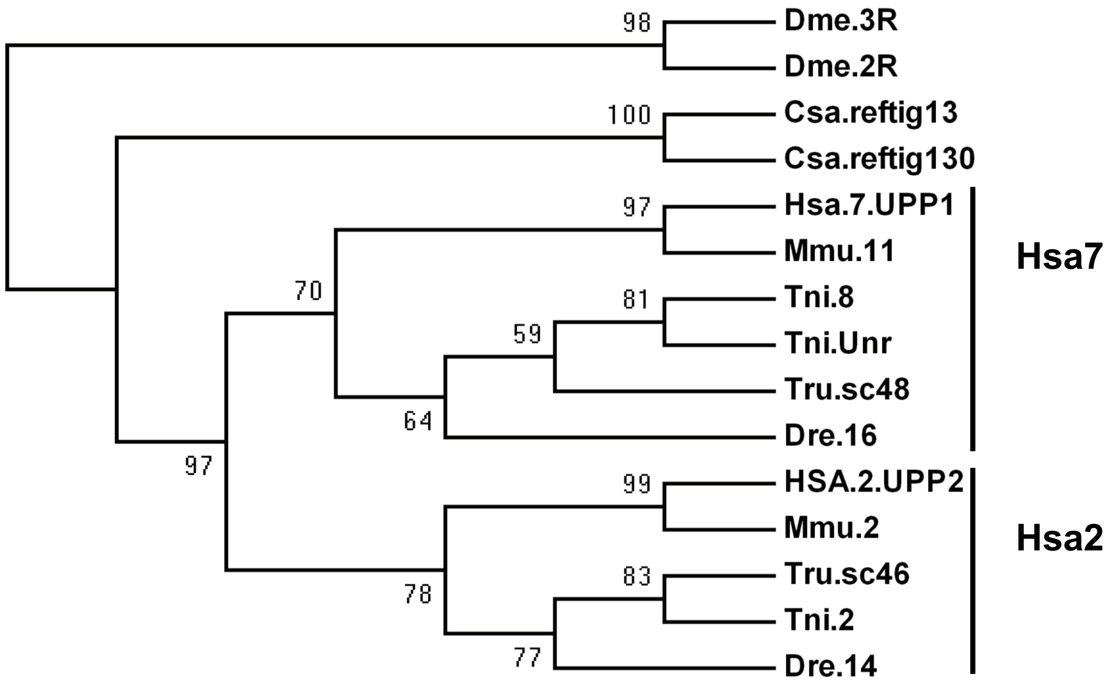

Phylogenetic tree showing the UPP family. Quartet-puzzling tree as implemented in Treepuzzle 5.2. Quartet-puzzling support values shown for each node. Abbreviations as in previous figures.

The tree was constructed using the following settings:
Parameter estimation: accurate (slow)
Parameter estimation uses: quartet sampling (for substitution process) + NJ tree (for rate variation)
Model of substitution: JTT
Amino acid frequencies estimated from data set
Model of rate heterogeneity: Gamma distributed rates
Gamma distribution parameter alpha estimated from data set
Number of Gamma rate categories: 8
Number of puzzling steps: 1000
